# Supplementary material for: All-visible-light-driven salicylidene schiff-base-functionalized artificial molecular motors
Source: Nat Commun. 2024 Jul 31;15:6461. doi: 10.1038/s41467-024-50587-4 (PMC11291758; doi:10.1038/s41467-024-50587-4)
Supplement: Supplementary file 1 — Supplementary Information [file 41467_2024_50587_MOESM1_ESM.pdf]

Supplementary Information

All-Visible-Light-Driven Salicylidene Schiff-Base-Functionalized Artificial Molecular Motors

Sven van Vliet[1,2,4], Jinyu Sheng[1,3,4], Charlotte N. Stindt[1] and Ben L. Feringa[1,*]

1: Stratingh Institute for Chemistry, University of Groningen, Groningen, the Netherlands.
2: Present address: Department of Energy Conversion and Storage, Technical University of Denmark, Kgs. Lyngby, Denmark.
3: Present address: Institute of Science and Technology Austria, Klosterneuburg, Austria.
4: These authors contributed equally: Sven van Vliet and Jinyu Sheng
Correspondence: b.l.feringa@rug.nl

Contents

All-Visible-Light-Driven Salicylidene Schiff-Base-Functionalized Artificial Molecular Motors

Supplementary Methods 3

1. Materials and characterization methods 3

2. Synthetic methods 4

Supplementary Discussion 9

3. UV-Vis analysis on the rotation of molecular motors 9

4. Eyring analysis on the thermal helix inversion of molecular motors. 11

5. Rotation of molecular motors in different solvents. 12

6. Rotary behaviour at different temperatures. 17

7. Characterization of rotary behaviour via ^{1}H NMR 22

8. Photoluminescence of motors 23

9. Photoisomerization of M4-Zn complex 24

10. Titration experiments of M4 with Zn^{2+} 25

11. Fatigue studies 25

12. Computational Analysis 27

13. NMR and HRMS spectra 28

Supplementary References 37

Supplementary Methods

1. Materials and characterization methods

Reagents were purchased from Sigma Aldrich, Acros or TCI Europe and used as received. Solvents were reagent-grade and used without prior water removal unless otherwise indicated. Anhydrous solvents were obtained from a solvent purification system (MBraun SPS-800). Flash column chromatography was performed on silica gel (Merck, type 9385, 230–400 mesh) or on a Büchi Reveleris purification system using Büchi silica cartridges. Thin layer chromatography (TLC) was carried out on aluminium sheets coated with silica gel 60 F254 (Merck). Compounds were visualised using a UV lamp (254 nm) and/or by staining with $KMnO_4$ or Cerium Ammonium Molybdate.

All reactions involving air sensitive reagents were performed under a N_2 atmosphere. ^1H and ^{13}C NMR spectra were recorded on a Varian Mercury-Plus 400 or a Bruker Avance 600 NMR spectrometer at 298 K unless otherwise indicated. PSS studies were performed on a Varian Unity Plus 500 NMR spectrometer. Chemical shifts are given in parts per million (ppm) relative to the residual solvent signal ($CDCl_3$ δ 7.26 for ^1H, δ 77.16 for ^{13}C, CD_2Cl_2 δ 5.32 for ^1H, δ 53.84 for ^{13}C and dimethylsulfoxide-d_6 δ 2.50 for ^1H, δ 39.52 for ^{13}C). Multiplets in ^1H NMR spectra are designated as follows: s (singlet), d (doublet), t (triplet), q (quartet), p (pentet), m (multiplet), br (broad). High resolution mass spectrometry (ESI+) was performed on an LTQ Orbitrap XL spectrometer. UV-Vis and CD spectra were recorded on a JASCO 810 CD spectrometer and performed in HPLC-graded solvents without degassing. High resolution mass spectrometry (ESI-MS) was performed on a LTQ Orbitrap XL spectrometer with ESI ionization. UV/Vis absorption spectra were measured on a Hewlett-Packard 8453 diode array spectrometer in a 1 cm quartz cuvette.

The UV-Vis and NMR irradiation experiments were performed using fiber-coupled LEDs (365, 405, 415, 455 nm and 470) obtained from Thorlabs Inc.

2. Synthetic methods

The parent Motor Z_{st}-**PM** (racemic and (*R,R*)-(*P,P*)) and Z_{st}-**PM**-OMe were synthesized using a literature protocol.[1]

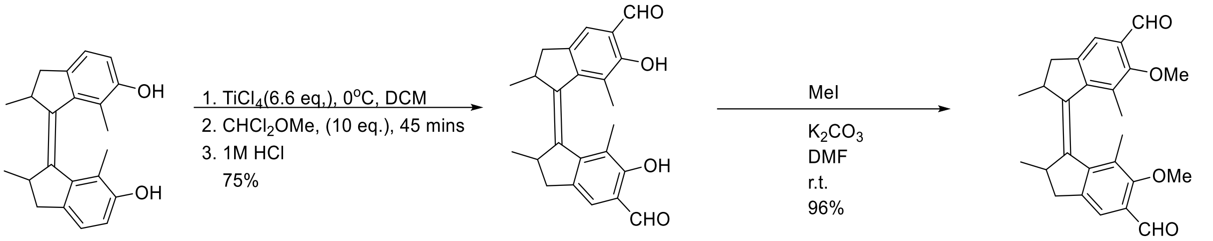

(*Z*)-6,6'-dihydroxy-2,2',7,7'-tetramethyl-2,2',3,3'-tetrahydro-[1,1'-biindenylidene]-5,5'-dicarbaldehyde

Under a N_2 atmosphere, diol-motor (192.0 mg, 0.6 mmol, 1.0 equiv.) was dissolved in dry DCM (10 mL). The solution was cooled to 0 °C and $TiCl_4$ (435.0 µL, 4.0 mmol, 6.6 equiv.) was slowly added forming a black suspension. After 20 min, dichloro(methoxy)methane (543.0 µL, 6 mmol, 10.0 equiv.) was added to the suspension and the color turned dark red. The mixture was slowly warmed to room temperature and stirred for another 45 min and then quenched with aq. 1 M HCl (10 mL). The mixture was extracted with EtOAc (3*25 mL) and the combined organic layers were washed with brine (150 mL), dried over Na_2SO_4 and concentrated under vacuum. The crude product was purified by column chromatography (SiO_2, pentane:EtOAc = 8:1 to 5:1) to afford Z_{st}-**PM** as yellow solid, further washed with HPLC grade pentane to obtain pure product (169.2 mg, 0.45 mmol, 75%). The same procedure was applied for the synthesis of the enantiomerically pure compound.

Note: The reaction mixture should be covered with Aluminum foil and all work-up steps should avoid long-time room light exposure as otherwise the compound will isomerize (as observed by ^{1}H NMR).

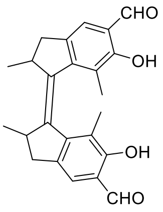

^{1}**H NMR** (400 MHz, CD_2Cl_2) δ 11.48 (s, 2H), 9.84 (s, 2H), 7.32 (s, 2H), 3.44 (p, *J* = 6.7 Hz, 2H), 3.24 (dd, *J* = 14.5, 6.3 Hz, 2H), 2.55 (d, *J* = 14.5 Hz, 2H), 1.40 (s, 6H), 1.10 (d, *J* = 6.7 Hz, 6H).
^{13}**C NMR** (101 MHz, $CDCl_3$) δ 196.0, 159.8, 149.2, 144.4, 136.4, 126.0, 124.7, 118.8, 42.8, 39.3, 20.0, 13.9.
HRMS (ESI pos) calcd $C_{24}H_{25}O_4$ $[M+H]^+$: 377.1747, found 377.1748.

(*Z*)-6,6'-dimethoxy-2,2',7,7'-tetramethyl-2,2',3,3'-tetrahydro-[1,1'-biindenylidene]-5,5'-dicarbaldehyde

Under a N_2 atmosphere, Z_{st}-**PM** (75.2 mg, 0.2 mmol, 1.0 equiv.) and K_2CO_3 (165.6 mg, 1.2 mmol, 6.0 equiv.) was dissolved in dry DMF (4.0 mL). Next MeI (0.1 mL, 1.6 mmol, 8.0 equiv.) was added to the suspension, the color of the red suspension slowly turned into slight yellow, and the mixture was stirred at room temperature for another 1 h. Water (20 mL) was added and the mixture extracted with EtOAc (3*25 mL). The combined organic layers were washed with brine (150 mL), dried over Na_2SO_4 and concentrated under vacuum. The crude yellow solid was washed with HPLC grade pentane to afford pure Z_{st}-**PM**-OMe as yellow solid (77.6 mg, 0.192 mmol, 96%). The same procedure was applied for the synthesis of the enantiomerically pure compound.

Note: The reaction mixture should be covered with Aluminum foil and all work-up steps should avoid long-time room light illumination. Otherwise, the compound will undergo isomerization.

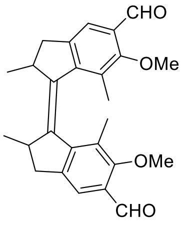

^{1}H NMR (400 MHz, $CDCl_3$) δ 10.37 (s, 2H), 7.63 (s, 2H), 3.85 (s, 7H), 3.43 (p, *J* = 6.9 Hz, 2H), 3.23 (dd, *J* = 14.9, 6.2 Hz, 2H), 2.55 (d, *J* = 14.8 Hz, 2H), 1.51 (s, 7H), 1.09 (d, *J* = 6.7 Hz, 7H).
^{13}C NMR (101 MHz, $CDCl_3$) δ 190.4, 161.3, 149.1, 144.0, 142.2, 129.8, 128.0, 121.7, 62.8, 43.0, 39.9, 20.0, 14.5.
HRMS (ESI pos) calcd $C_{26}H_{29}O_4$ $[M+H]^+$: 405.2060, found 405.2058.

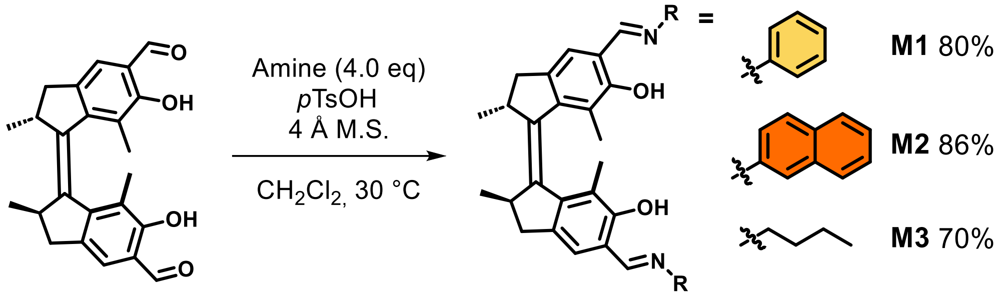

Parent Motor **PM**
Racemic

General procedure salicylidene Schiff base formation:
To a Schlenk flask containing the parent motor Z_{st}-**PM**[1] (1.0 eq) was added anhydrous CH_2Cl_2 (0.03 M), and the tube was covered in aluminium foil in order to protect the solution from light. The desired amine (6.0 eq) was added, together with *p*TsOH (0.05 eq) and a spatula tip of powdered 4 Å molecular sieves and the reaction mixture was heated at 30 °C. After completion of the reaction, monitored by TLC, the solvent was removed under reduced pressure and the crude solid was purified by column chromatography (SiO_2; pentane : EtOAc 9 : 1) to obtain the desired Z_{st}-salicylidene Schiff base-functionalized motor.

(*Z*)-2,2',7,7'-tetramethyl-5,5'-bis((*E*)-(phenylimino)methyl)-2,2',3,3'-tetrahydro-[1,1'-biindenylidene]-6,6'-diol (Z_{st}-M1)

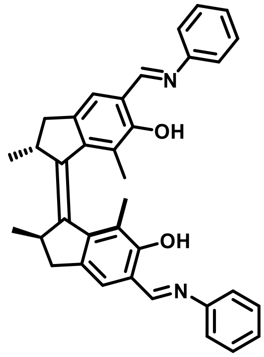

Starting from *Z*-**PM** (23 mg, 0.061 mmol), the title compound was obtained as orange solid (26 mg, 80%).

^{1}H NMR (400 MHz,CD_2Cl_2) δ 13.76 (br, 2H), 8.66 (br, 2H), 7.43 (m, 4H), 7.30 (m, 6H), 7.23 (s, 2H), 3.38 (m, 2H), 3.21 (dd, J = 14.3, 5.4 Hz, 2H), 2.50 (d, J = 14.3 Hz, 2H), 1.42 (s, 6H), 1.07 (d, J = 7.1 Hz, 6H).
^{13}C NMR (101 MHz, $CDCl_3$) δ 162.64, 158.95, 148.96, 146.12, 143.32, 135.77, 129.52, 126.64, 125.13, 124.99, 124.35, 121.29, 117.24, 77.41, 42.75, 39.55, 20.25, 14.31.
HRMS (ESI+, m/z) calcd for $C_{36}H_{34}N_2O_2$ $[M+H^+]$= 527.2693, found 527.2699.

(*Z*)-2,2',7,7'-tetramethyl-5,5'-bis((*E*)-(naphthalen-2-ylimino)methyl)-2,2',3,3'-tetrahydro-[1,1'-biindenylidene]-6,6'-diol (Z_{st}-M2)

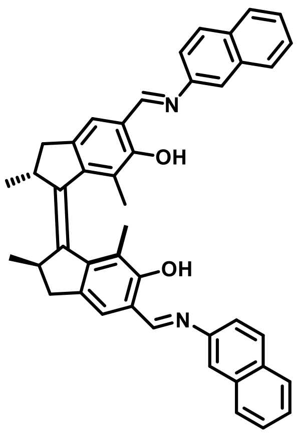

Starting from *Z*-**PM** (50 mg, 0.13 mmol), the title compound was obtained as orange solid (72 mg, 86%).

^{1}H NMR (400 MHz,CD_2Cl_2) δ 13.74 (br, 2H), 8.82 (br, 2H), 7.94 – 7.87 (m, 7H), 7.75 (m, 2H), 7.56 – 7.48 (m, 7H), 7.27 (s, 2H), 3.44 (p, J = 6.6 Hz, 2H), 3.27 (dd, J = 14.4, 6.6 Hz, 2H), 2.55 (d, J = 14.4 Hz, 2H), 1.13 (d, J = 6.6 Hz, 6H).
^{13}C NMR (101 MHz, $CDCl_3$) δ 162.46, 158.89, 146.27, 146.05, 143.24, 135.69, 134.03, 132.19, 129.26, 128.00, 127.76, 126.60, 125.69, 124.88, 120.62, 118.66, 117.23, 77.21, 42.60, 39.41, 20.11, 14.18.
Note: due to extreme light sensibility of Z_{st}-**M2** the sample contains ca. 20% E_{st}-**M2**.
HRMS (ESI+, m/z) calcd for $C_{44}H_{38}N_2O_2$ [M+H$^+$]= 627.3006, found 627.3023.

(*Z*)-5,5'-bis((*E*)-(butylimino)methyl)-2,2',7,7'-tetramethyl-2,2',3,3'-tetrahydro-[1,1'-biindenylidene]-6,6'-diol (Z_{st}-M3)

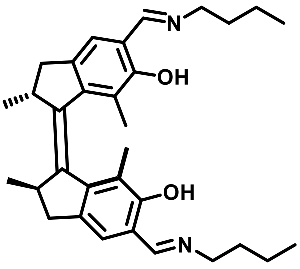

Starting from *Z*-**PM** (50 mg, 0.13 mmol), the title compound was obtained as orange solid (46 mg, 70%).

^{1}H NMR (400 MHz, CD_2Cl_2) δ 13.83 (br, 2H), 8.32 (s, 2H), 7.03 (s, 2H), 3.64 – 3.56 (m, 4H), 3.37 (p, J = 6.4 Hz, 2H), 3.19 (dd, J = 14.1, 6.4 Hz, 2H), 2.46 (d, J = 14.1 Hz, 2H), 1.72 – 1.64 (m, 4H), 1.46 – 1.41 (m, 10H), 1.08 (d, J = 6.4 Hz, 6H), 0.96 (t, J = 7.3 Hz, 6H).
^{13}C NMR (101 MHz, CD_2Cl_2) δ 164.42, 159.92, 144.81, 142.54, 134.74, 124.05, 123.64, 116.39, 58.93, 42.50, 39.42, 33.04, 20.31, 20.06, 14.07, 13.80.
HRMS (ESI+, m/z) calcd for $C_{32}H_{42}N_2O_2$ [M+H$^+$]= 487.3319, found 487.3324.

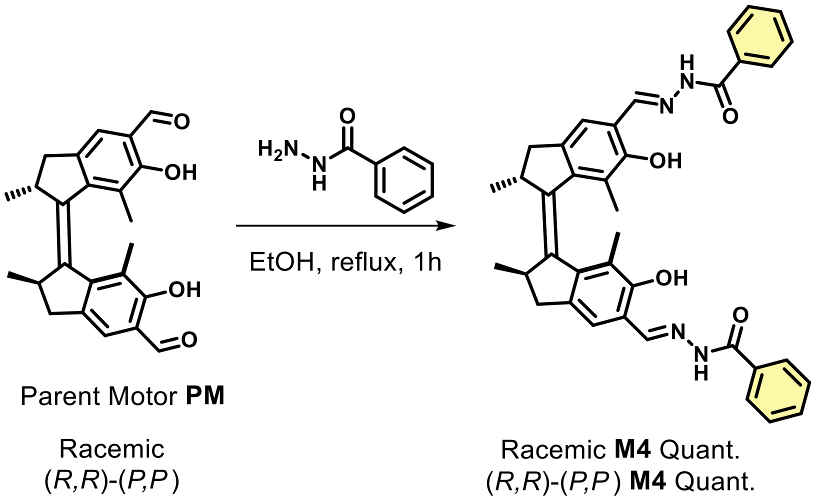

Parent Motor **PM**

Racemic
(*R,R*)-(*P,P*)

Racemic **M4** Quant.
(*R,R*)-(*P,P*) **M4** Quant.

Synthetic procedure for Z_{st}**-M4:**
The parent motor Z_{st}-**PM**[1] (20 mg, 0.053 mmol, 1.0 eq) was dissolved in EtOH (1 mL) in an aluminium foil covered round bottom flask. Benzhydrazide (14 mg, 0.11 mmol, 2.0 eq) was added and the mixture was heated at reflux for 1 h. Hereafter, the reaction mixture was allowed to cool to room temperature, the precipitate was filtered off and washed with ice cold EtOH (2 x 2 mL) to obtain Z_{st}-**M4** as bright yellow solid (32 mg, quant.)

***N',N'''*-((1E,1'E)-(*Z*)-6,6'-dihydroxy-2,2',7,7'-tetramethyl-2,2',3,3'-tetrahydro-[1,1'-biindenylidene]-5,5'-diyl)bis(methaneylidene))di(benzohydrazide) (*Z*$_{st}$-M4)**

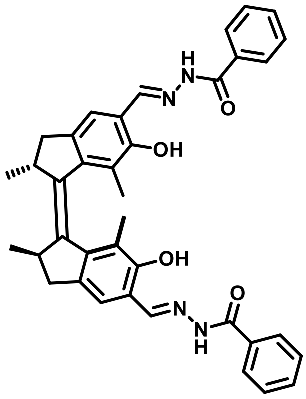

^{1}H NMR (400 MHz, DMSO-d$_6$) δ 12.18 (br, 2H), 11.92 (br, 2H), 8.57 (br, 2H), 7.94 (dd, J = 7.0, 1.8 Hz, 4H), 7.65 – 7.58 (m, 2H), 7.57 – 7.50 (m, 4H), 7.22 (s, 2H), 3.17 (dd, J = 13.9, 6.1 Hz, 2H), 1.39 (s, 6H), 1.06 (d, J = 6.7 Hz, 6H).
^{13}C NMR (101 MHz, DMSO-d$_6$) δ 163.09, 155.35, 150.51, 144.22, 142.47, 136.13, 133.10, 132.51, 129.04, 128.10, 124.20, 123.03, 116.38, 42.48, 40.61, 40.44, 40.10, 20.48, 14.58.
HRMS (ESI+, m/z) calcd for $C_{38}H_{36}N_4O_4$ [M+H$^+$]= 613.2809, found 613.2809.

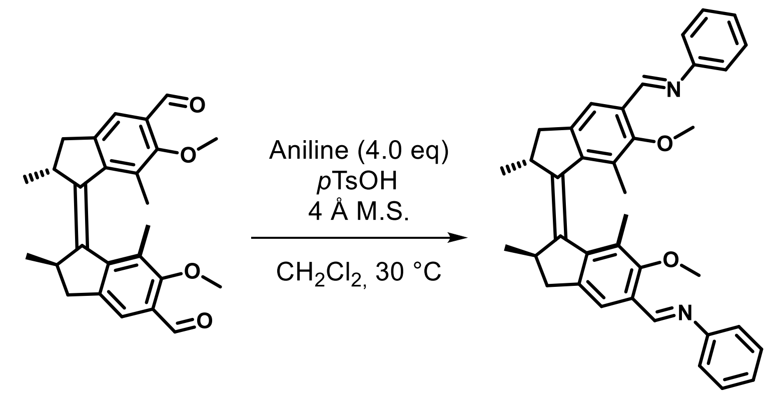

Parent Motor **PM**-OMe
Racemic

Racemic **M1**-OMe

Synthetic procedure for Z_{st}-M1-OMe:
To a Schlenk flask containing the parent motor Z_{st}-**PM**-OMe[1] (1.0 eq) was added anhydrous CH_2Cl_2 (0.03 M) and the tube was covered in aluminium foil in order to protect the solution from light. Aniline (6.0 eq) was added, together with *p*TsOH (0.05 eq) and a spatula tip of powdered 4 Å molecular sieves and the reaction mixture was heated at 30 °C. After completion of the reaction, monitored by TLC, the solvent was removed under reduced pressure. It was attempted to further purify the crude solid by column chromatography (SiO_2; pentane : EtOAc 9 : 1) in two consecutive cycles. However, due to the rapid hydrolysis of Z_{st}-**M1**-OMe at ambient conditions, **M1**-OMe lacks the stabilizing intramolecular H-bonding effect, it was opted to directly use Z_{st}-**M1**-OMe without further purification.

(1E,1'E)-1,1'-((2R,2'R,Z)-6,6'-dimethoxy-2,2',7,7'-tetramethyl-2,2',3,3'-tetrahydro-[1,1'-biindenylidene]-5,5'-diyl)bis(N-phenylmethanimine) (M1-OMe)

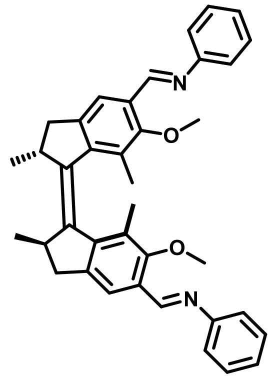

^{1}H NMR (400 MHz, $CDCl_3$) δ 8.83 (s, 2H), 7.97 (s, 2H), 7.45 – 7.40 (m, 4H), 7.26 – 7.22 (m, 6H), 3.79 (s, 6H), 3.43 (p, J = 6.7, 4.1 Hz, 2H), 3.24 (dd, J = 5.6, 3.9 Hz, 2H), 2.54 (d, *J* = 5.8 Hz, 2H), 1.53 (s, 6H), 1.10 (d, J = 6.7 Hz, 6H).
HRMS (ESI+, m/z) calcd for $C_{38}H_{38}N_2O_2$ [M+H$^+$]= 555.3006, found 555.2985.

Supplementary Discussion

3. UV-Vis analysis on the rotation of molecular motors

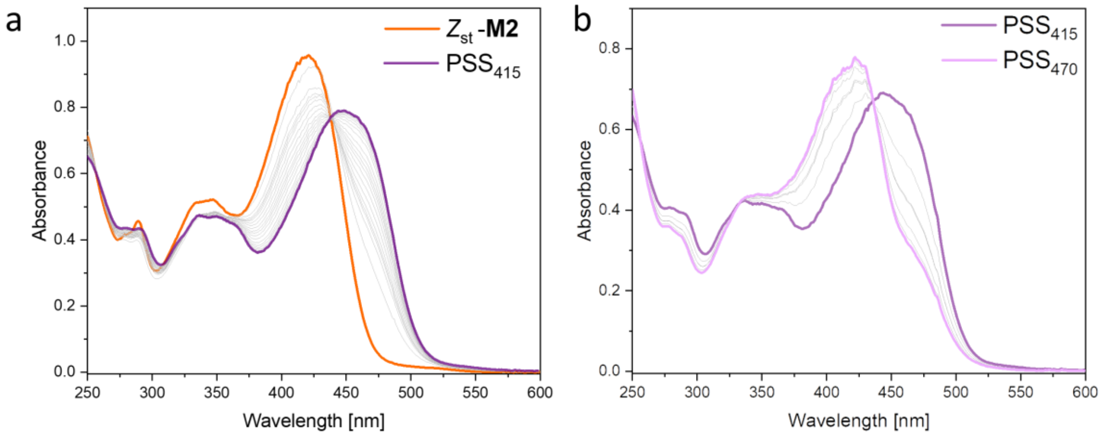

Supplementary Figure 1. **Photoisomerization behaviour of M2**. **a** UV-Vis spectra of **M2** at PSS (Z_{st}-**M2** → Z_{mst}-**M2**) upon 415 nm light irradiation (purple). **b** UV-Vis spectra of **M2** at PSS (Z_{mst}-**M2** → E_{st}-**M2**, fuchsia) upon 470 nm light irradiation measured in *i*-PrOH.

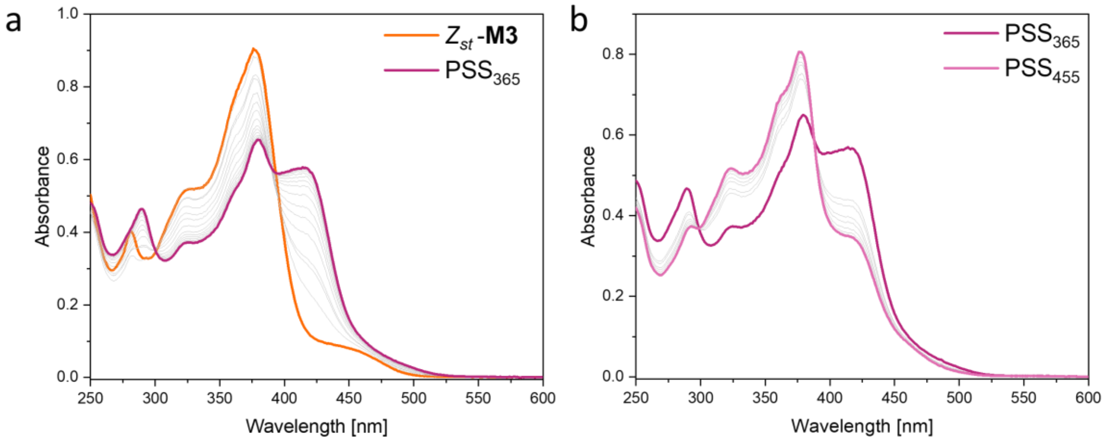

Supplementary Figure 2. **Photoisomerization behaviour of M3.** **a** UV-Vis spectra of **M3** at PSS (Z_{st}-**M3** → Z_{mst}-**M3**) upon 365 nm light irradiation (purple). **b** UV-Vis spectra of **M3** at PSS (Z_{mst}-**M3** → E_{st}-**M3**, pink) upon 455 nm light illumination measured in *i*-PrOH.

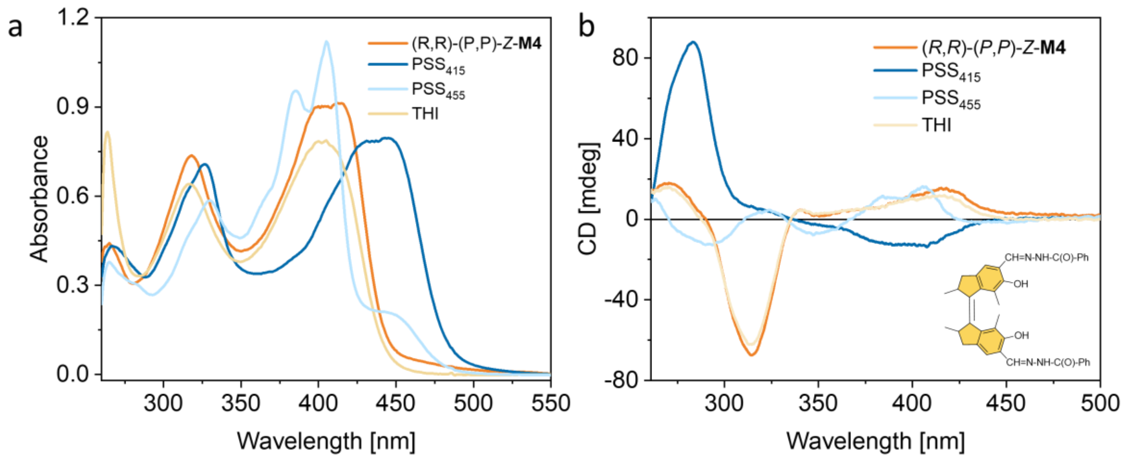

Supplementary Figure 3. **Photoisomerization behaviour of M4. a** UV-Vis spectra (DMSO, 293 K) of (*R,R*)-(*P*,*P*)-*Z*-**M4** at four different states. **b** CD spectra of (*R,R*)-(*P*,*P*)-*Z*-**M4** at four different states. Note: spectra of **M4** were measured in DMSO due to solubility issues.

4. Eyring analysis on the thermal helix inversion of molecular motors.

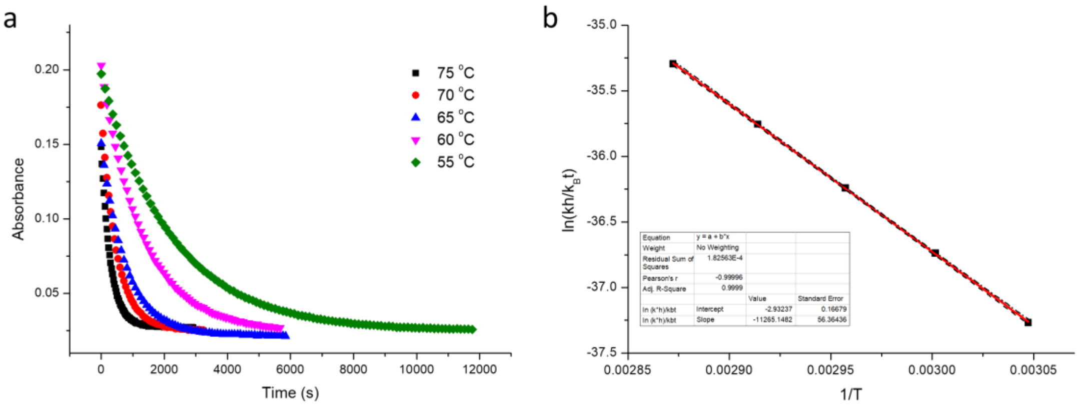

Supplementary Figure 4. **Activation barriers of M1**. **a** Absorbance decay of Z_{mst}-**M1** at different temperatures. **b** Linearized Eyring plot analysis on the THI of metastable Z_{mst}-**M1** in *i*-PrOH. Dotted lines represent 95% confidence intervals.

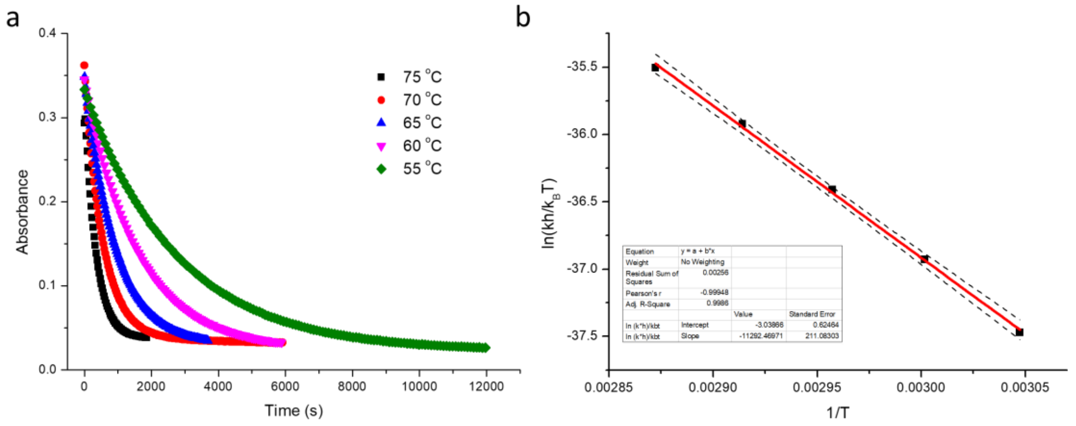

Supplementary Figure 5. **Activation barriers of M2**. **a** Absorbance decay of Z_{mst}-**M2** at different temperatures. **b** Linearized Eyring plot analysis on the THI of metastable Z_{mst}-**M2** in *i*-PrOH. Dotted lines represent 95% confidence intervals.

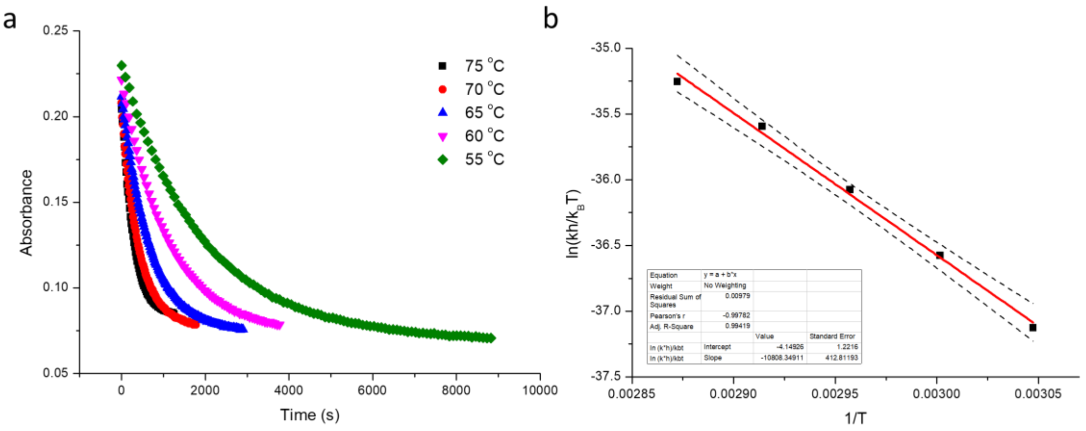

Supplementary Figure 6. **Activation barriers of M3**. **a** Absorbance decay of *Z*$_{mst}$-**M3** at different temperatures. **b** Linearized Eyring plot analysis on the THI of metastable *Z*$_{mst}$-**M3** in *i*-PrOH. Dotted lines represent 95% confidence intervals.

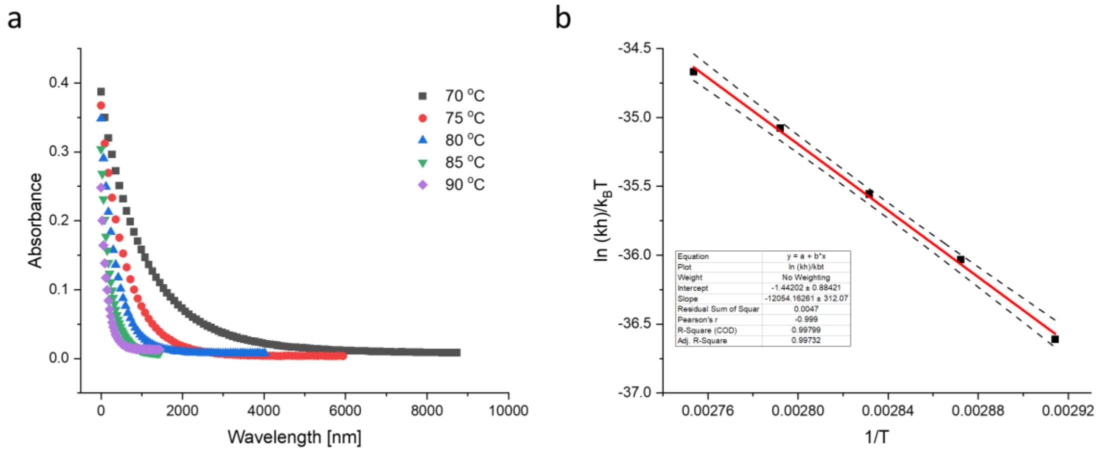

Supplementary Figure 7. **Activation barriers of M4**. **a** Absorbance decay of *Z*$_{mst}$-**M4** at different temperatures. **b** Linearized Eyring plot analysis on the THI of metastable *Z*$_{mst}$-**M4** in DMSO. Dotted lines represent 95% confidence intervals.

5. Rotation of molecular motors in different solvents.

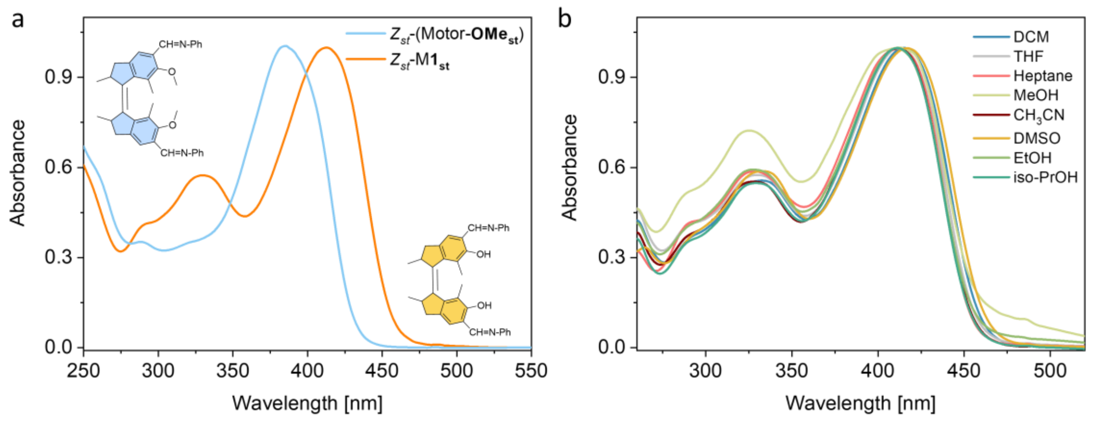

Supplementary Figure 8. **Absorbance of Motors in different solvents. a** normalized UV-Vis spectra of *Z*$_{st}$-**M1** and Z$_{st}$-**M1-OMe** for comparison (*i*-PrOH). **b** Normalized UV-Vis spectra of *Z*$_{st}$-**M1** in a range of solvents.

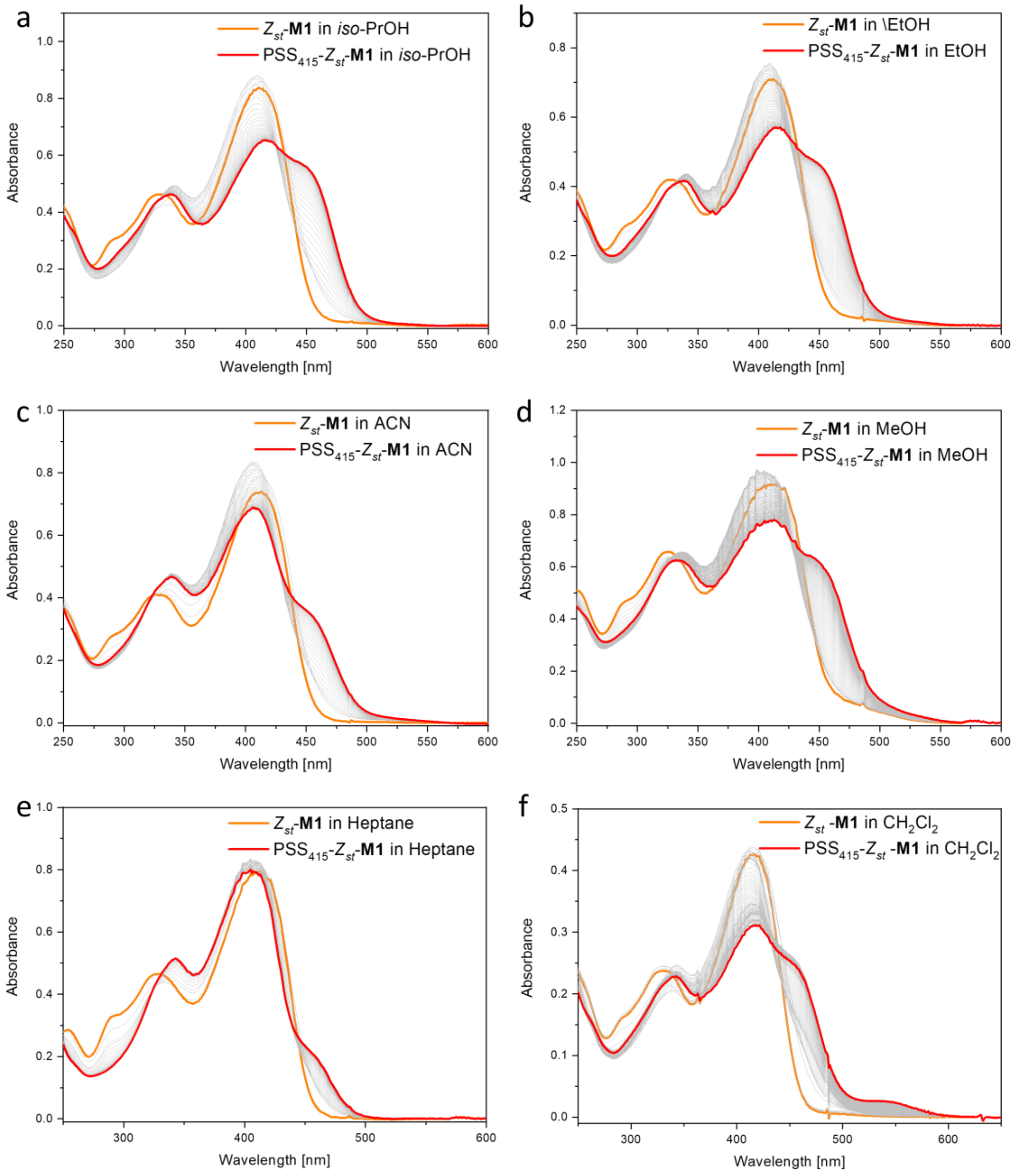

Supplementary Figure 9. **UV-Vis spectra of M1 at PSS upon 415 nm light irradiation at 293 K (*Z_{st}*-M1 → *Z_{mst}*-M1 isomerization) in different solvents.** (**a** isopropanol; **b** ethanol; **c** acetonitrile; **d** methanol; **e** heptane and **f** CH_2Cl_2.)

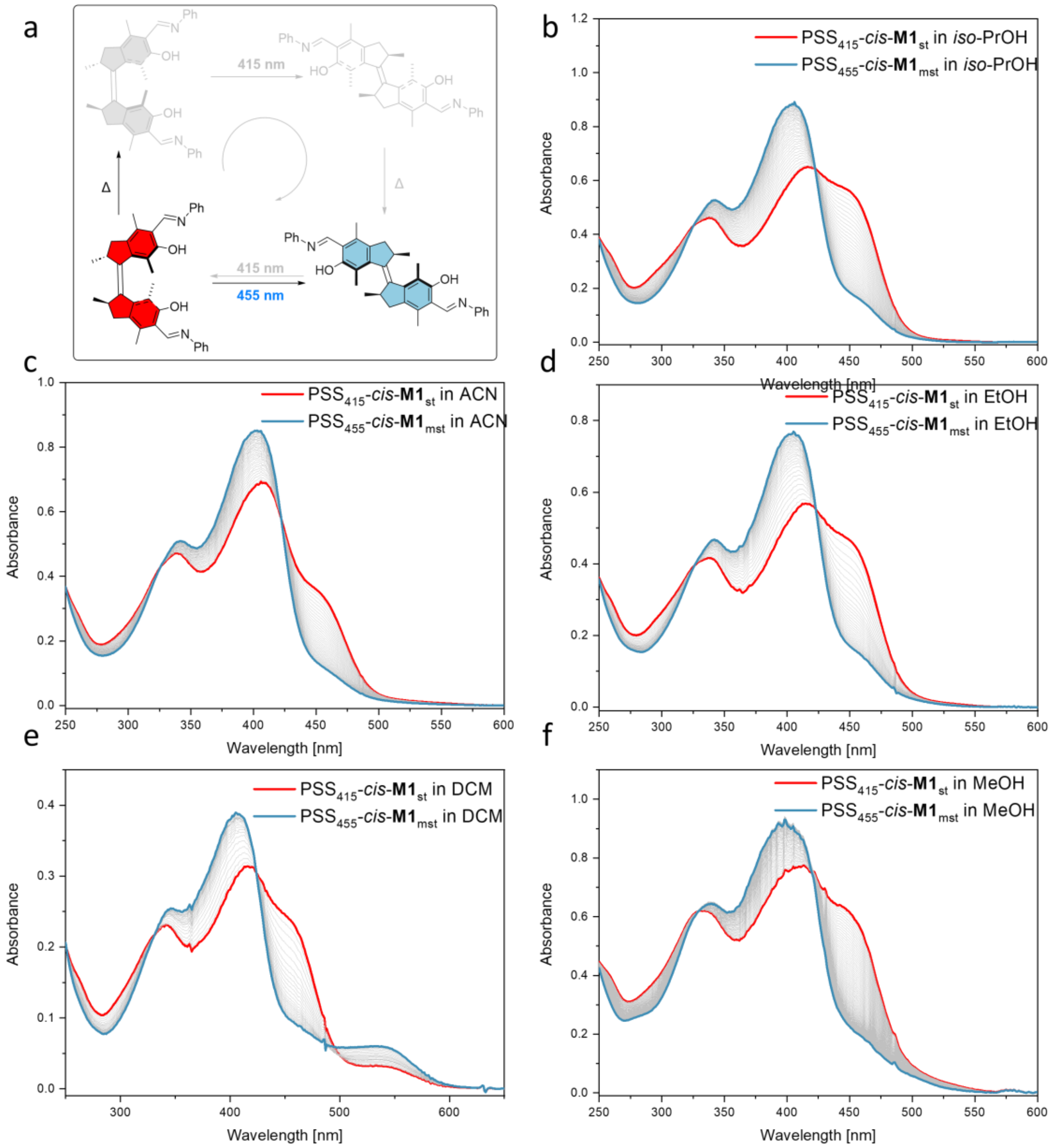

Supplementary Figure 10. **UV-Vis spectra of M1 at PSS upon 455 nm light irradiation at 293 K (*Z*$_{mst}$-M1 → *E*$_{st}$-M1 isomerization) in different solvents**. (**a** rotary scheme of **M1**. **b** isopropanol; **c** acetonitrile; **d** ethanol; **e** CH_2Cl_2 and **f** methanol.)

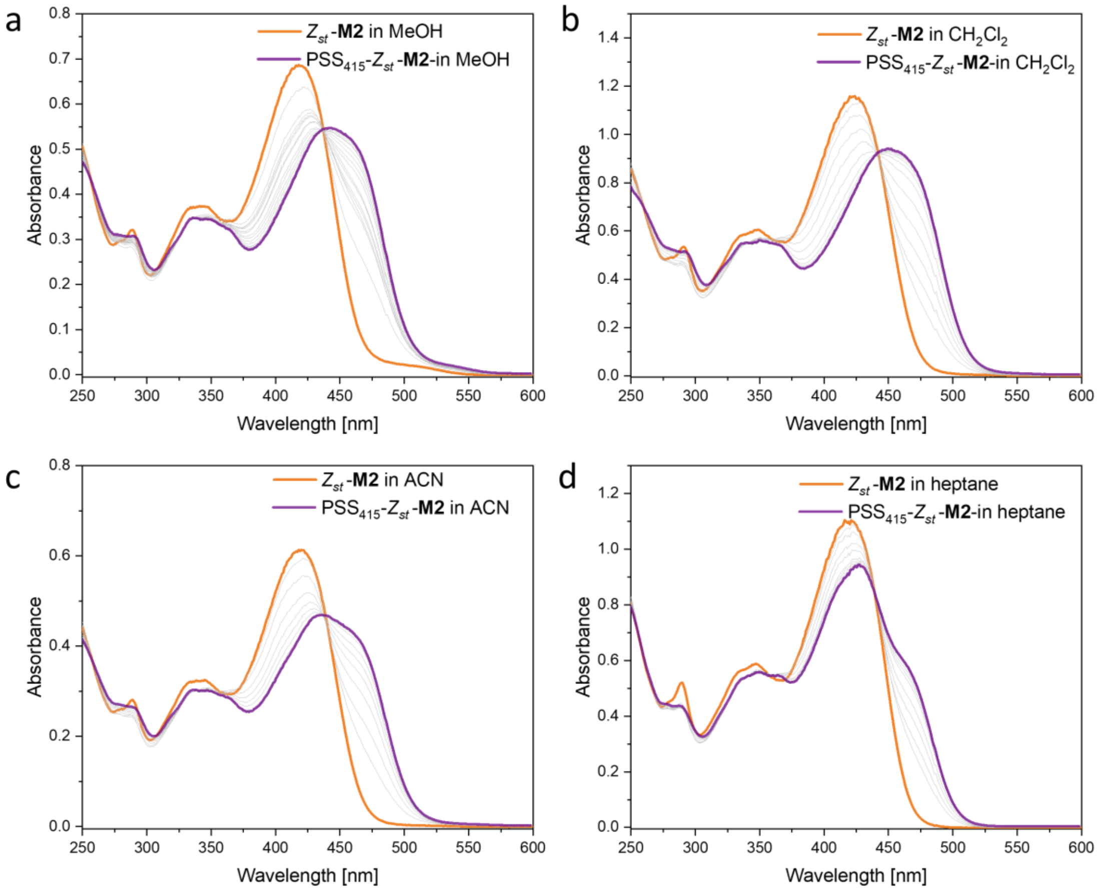

Supplementary Figure 11. UV-Vis spectra of M2 at PSS upon 415 nm light irradiation at 293 K (Z_{st}-M2 → Z_{mst}-M2 isomerization) in different solvents. (**a** methanol; **b** CH_2Cl_2; **c** acetonitrile and **d** heptane.)

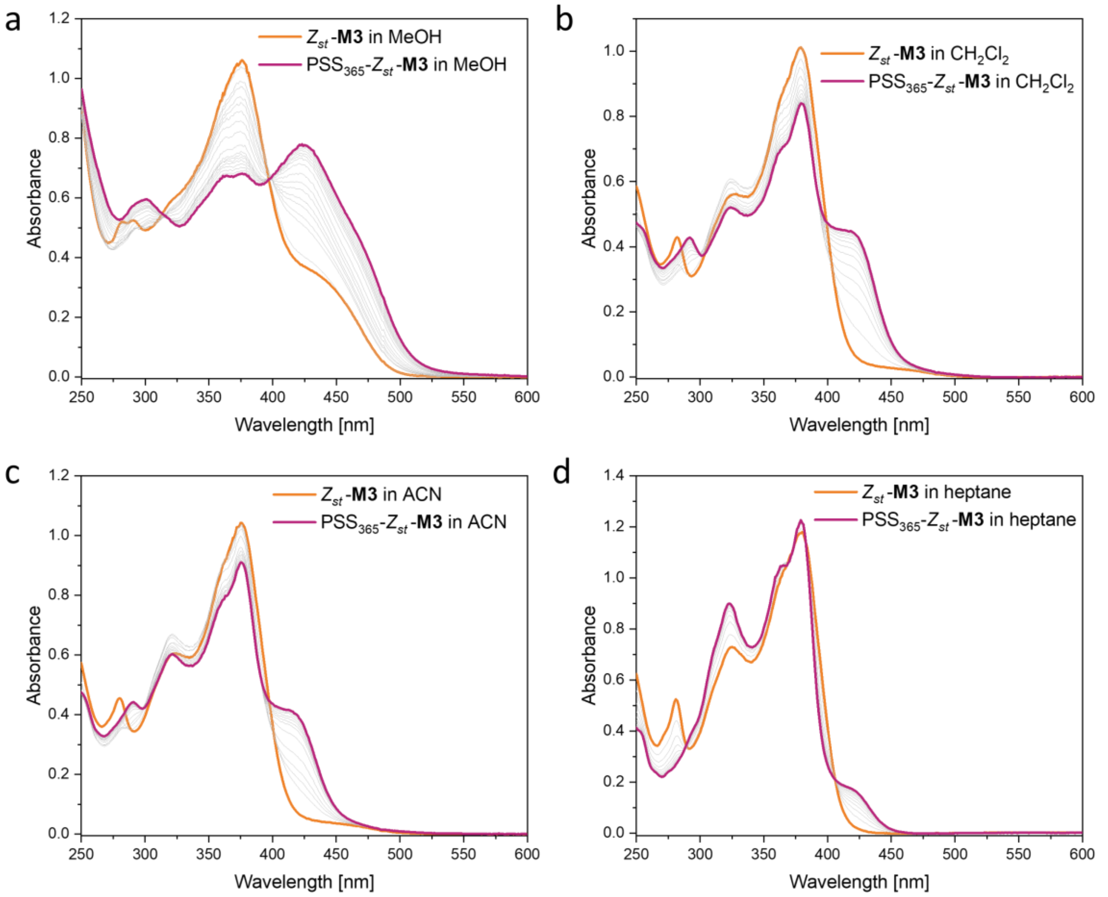

Supplementary Figure 12. **UV-Vis spectra of M3 at PSS upon 365 nm light irradiation at 293 K (*Z_{st}*-M3 → *Z_{mst}*-M3 isomerization) in different solvents.** (**a** methanol; **b** CH_2Cl_2; **c** acetonitrile and **d** heptane.)

6. Rotary behaviour at different temperatures.

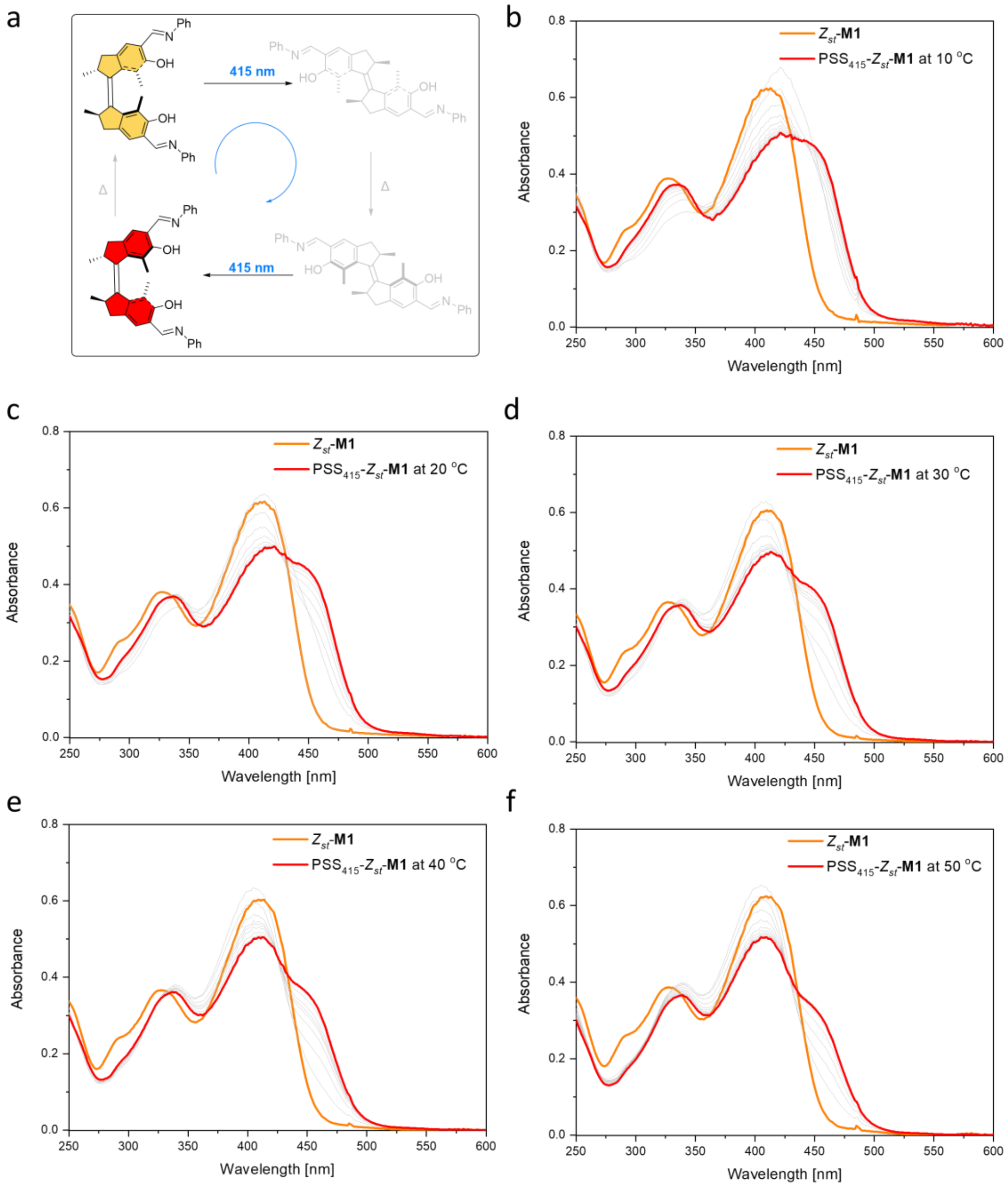

Supplementary Figure 13. UV-Vis spectra of M1 at PSS upon 415 nm light irradiation (Z_{st}-M1 → Z_{mst}-M1 isomerization) at different temperatures. (**a** rotary scheme of **M1**. **b** to **f** from 10 °C to 50 °C.)

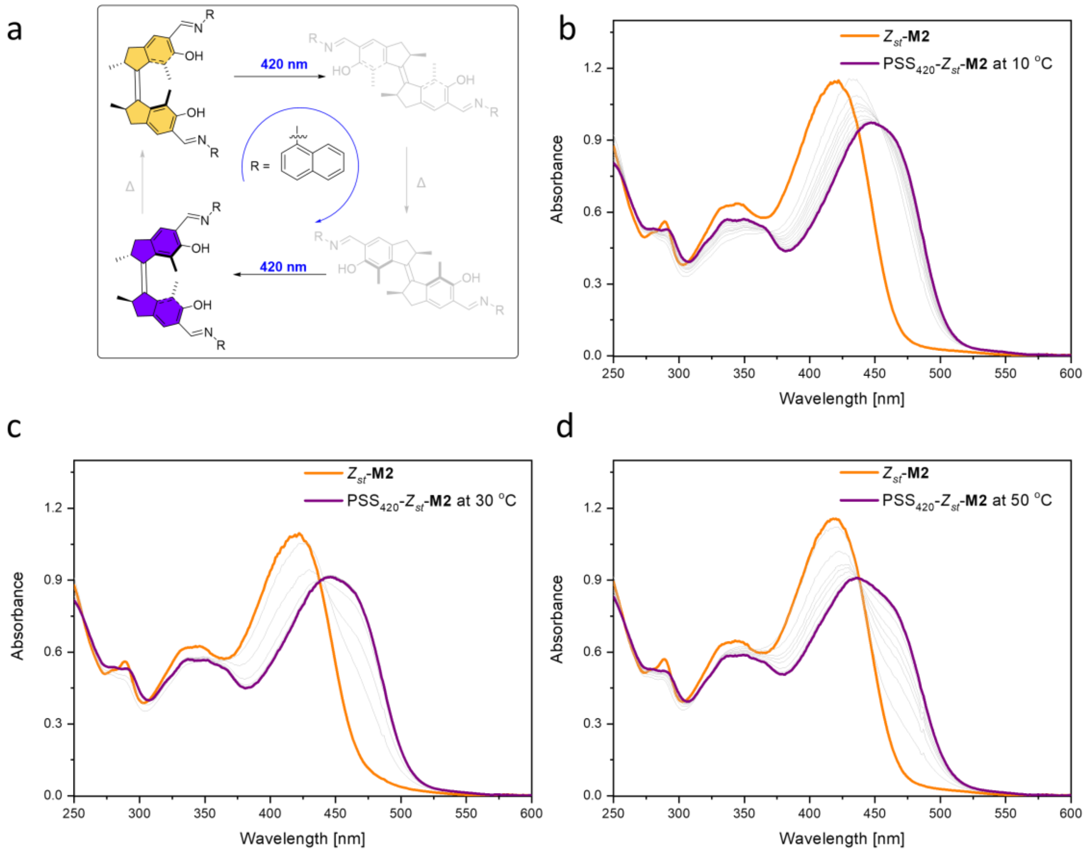

Supplementary Figure 14. **UV-Vis spectra of M2 at PSS upon 420 nm light irradiation (*Z_{st}*-M2 → *Z_{mst}*-M2 isomerization) at different temperatures.** (**a** rotary scheme of **M2**. **b** to **d** from 10 °C to 50 °C.)

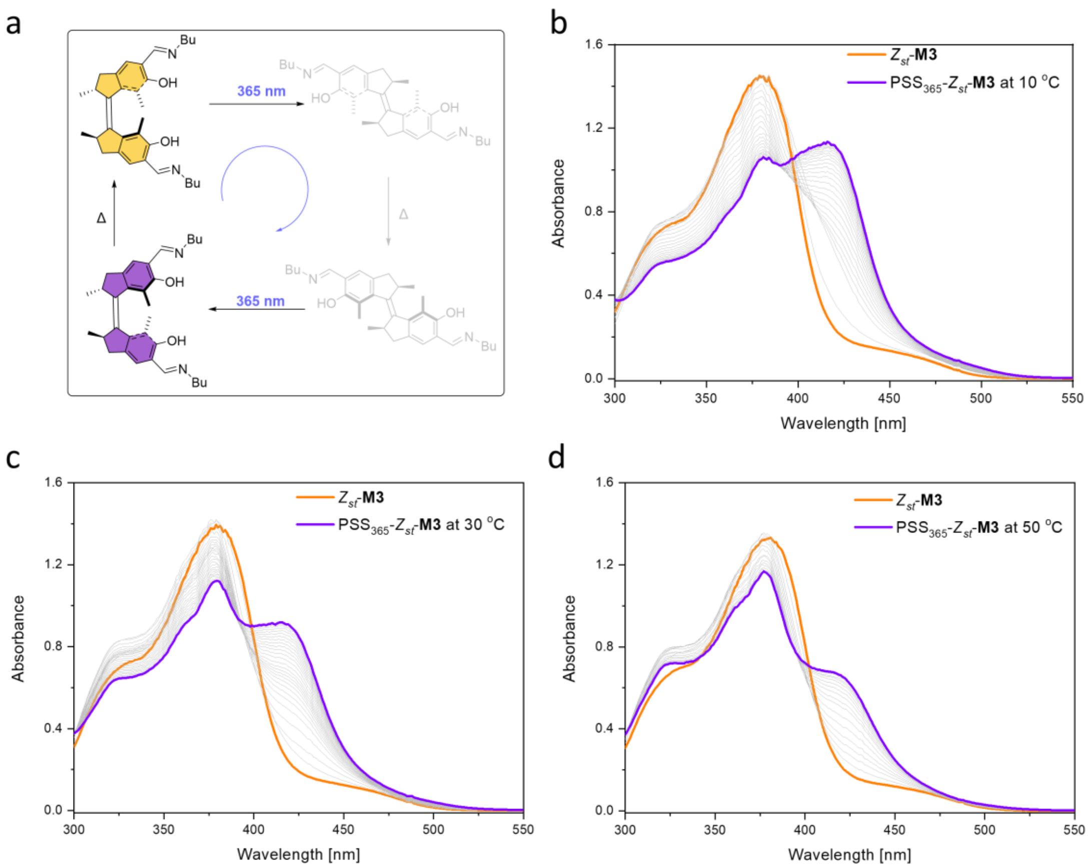

Supplementary Figure 15. UV-Vis spectra of M3 at PSS upon 365 nm light irradiation (*Z*$_{st}$-M3 → *Z*$_{mst}$-M3 isomerization) at different temperatures. (**a** rotary scheme of **M3**. **b** to **d** from 10 °C to 50 °C.)

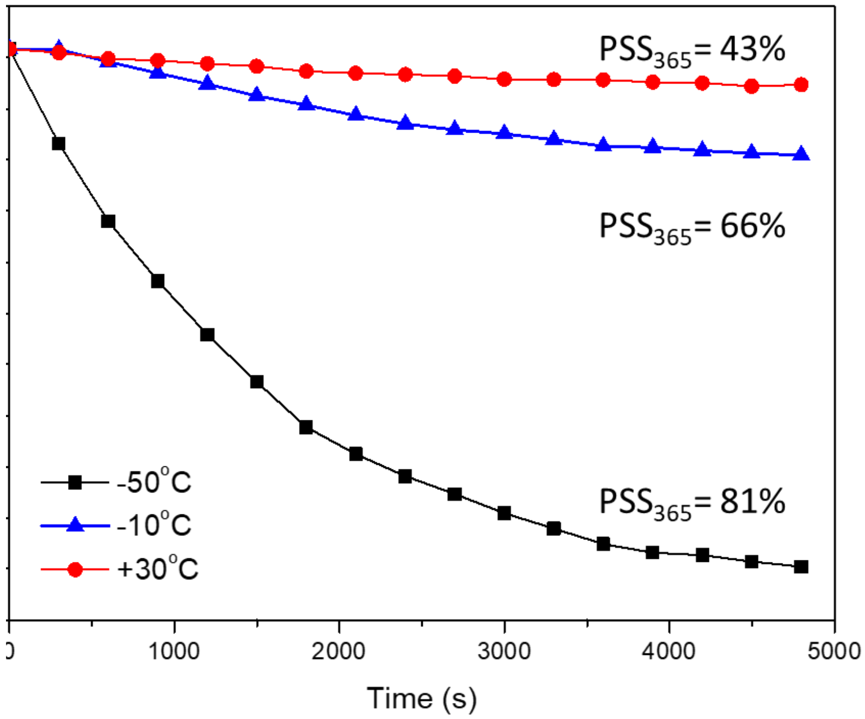

Supplementary Figure 16. PSS (*E$_{st}$*-M3 → *Z$_{mst}$*-M3) at different temperatures based on ^{1}H NMR analysis. Note: At room temperature, the first photochemical step **from** *Z$_{st}$*-**M** → *E$_{mst}$*-**M** followed by subsequent THI makes the first step photochemical step almost quantitative. However, at low temperature (-50 °C) the THI step, i.e. *E$_{mst}$*-M → *E$_{st}$*-M, is extremely slow, so enabling us to investigate the temperature-dependent PSS from *E$_{st}$*-**M** → *Z$_{mst}$*-**M.**

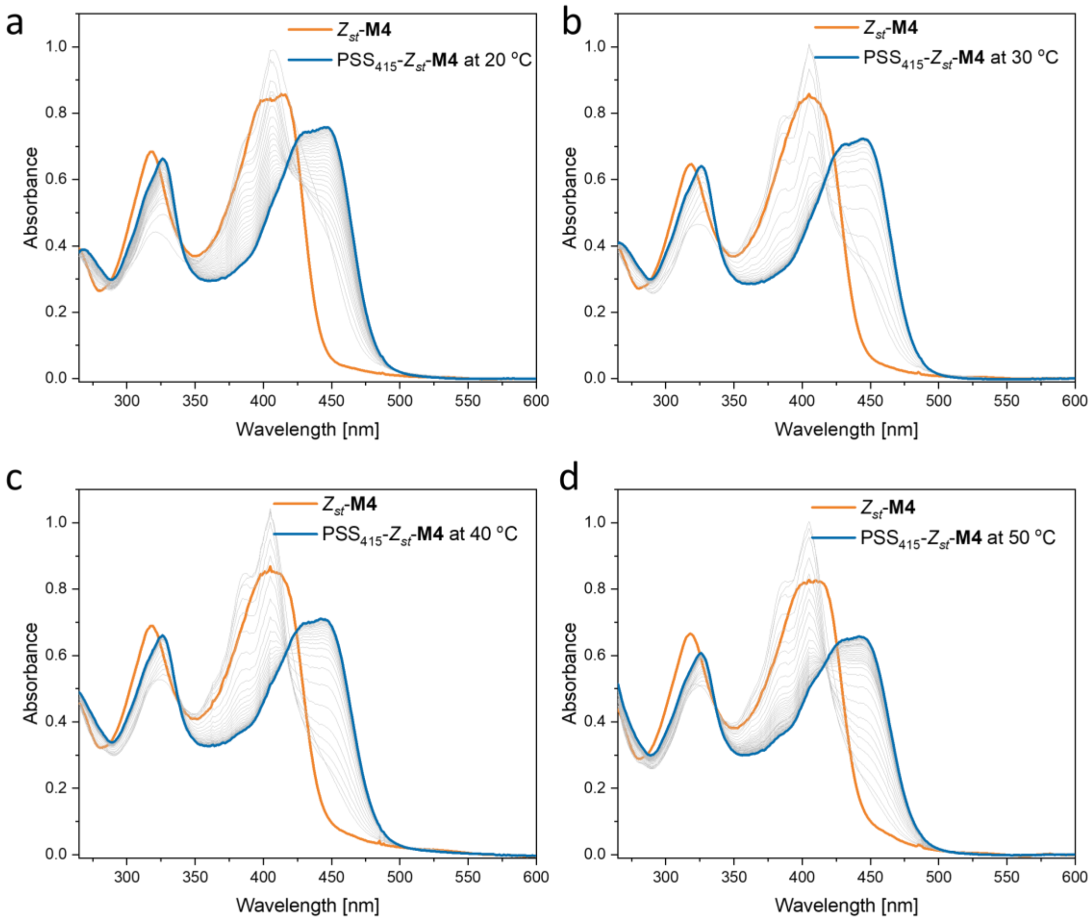

Supplementary Figure 17. **UV-Vis spectra of M4 at PSS upon 415 nm light irradiation (*Z_{st}*-M4 → *Z_{mst}*-M4 isomerization)**. Temperatures increase in **a** to **d** from 20 to 50 °C, indicating **M4** does not show temperature depend PSS phenomena.

7. Characterization of rotary behaviour via ^{1}H NMR

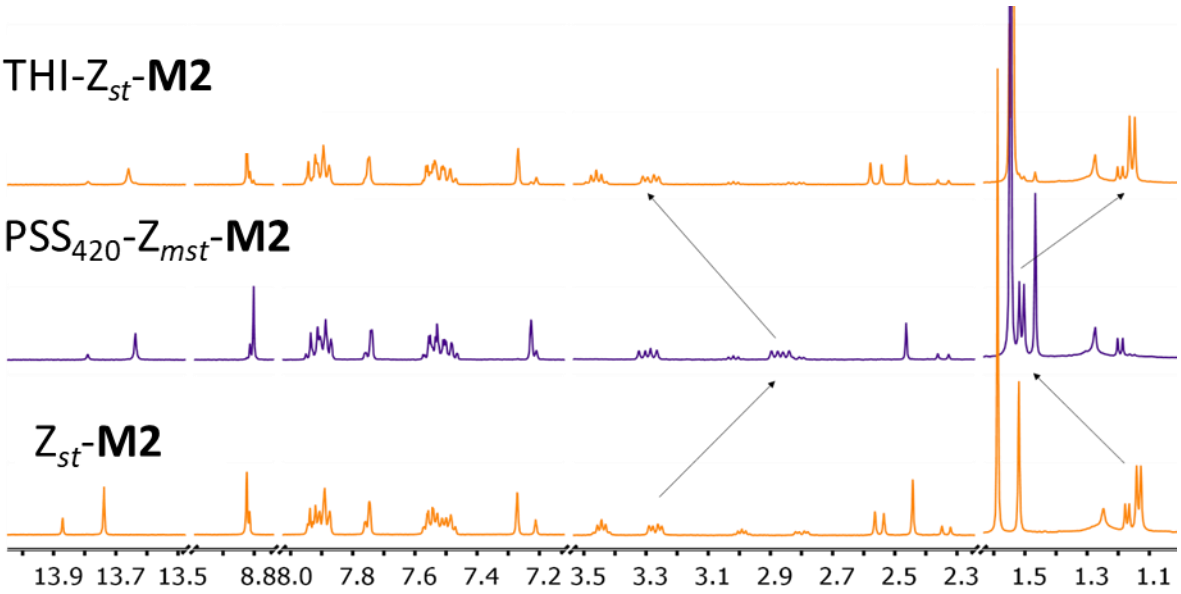

Supplementary Figure 18. Rotary behaviour of M2 investigated by ^{1}H NMR (CD_2Cl_2, 293 K): before irradiation at 293 K (orange, bottom), at PSS (purple, middle) and after THI (orange, top). Note: the initial spectrum (bottom) contains ca. 20% of E_{st}-**M2** due to extreme light sensibility.

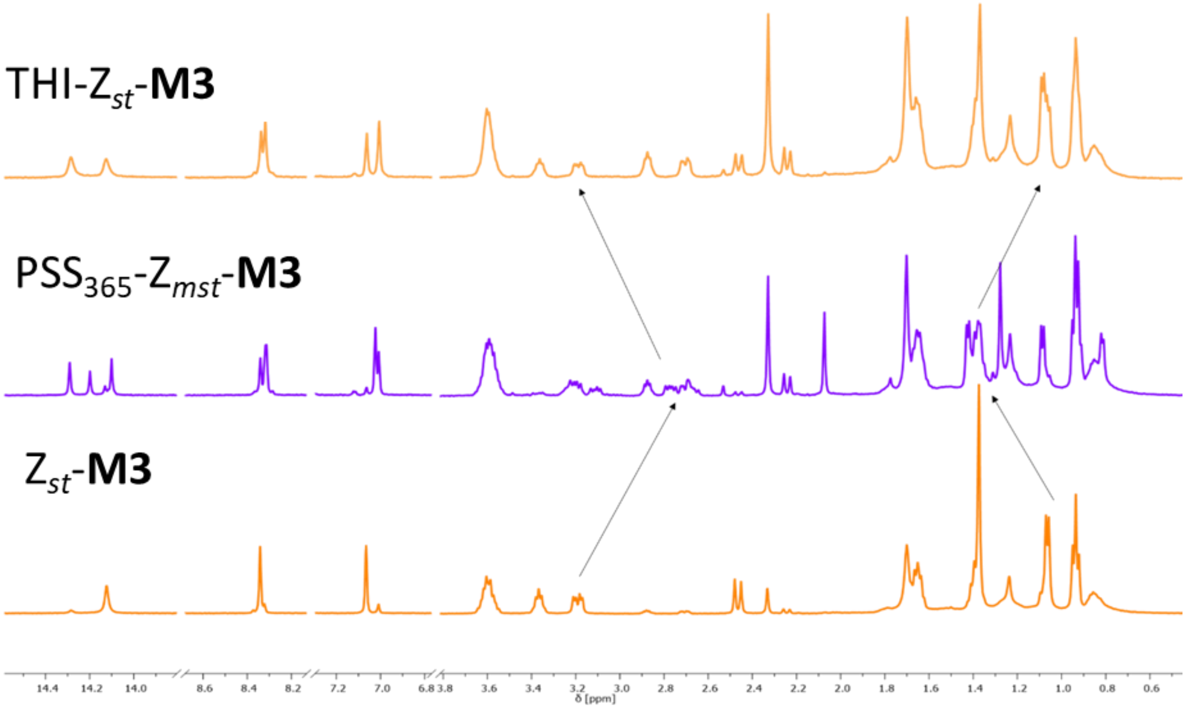

Supplementary Figure 19. Rotary behaviour of M3 investigated by ^{1}H NMR (CD_2Cl_2, 293 K): before irradiation at 293 K (orange, bottom), at PSS (purple, middle) and after THI (orange, top).

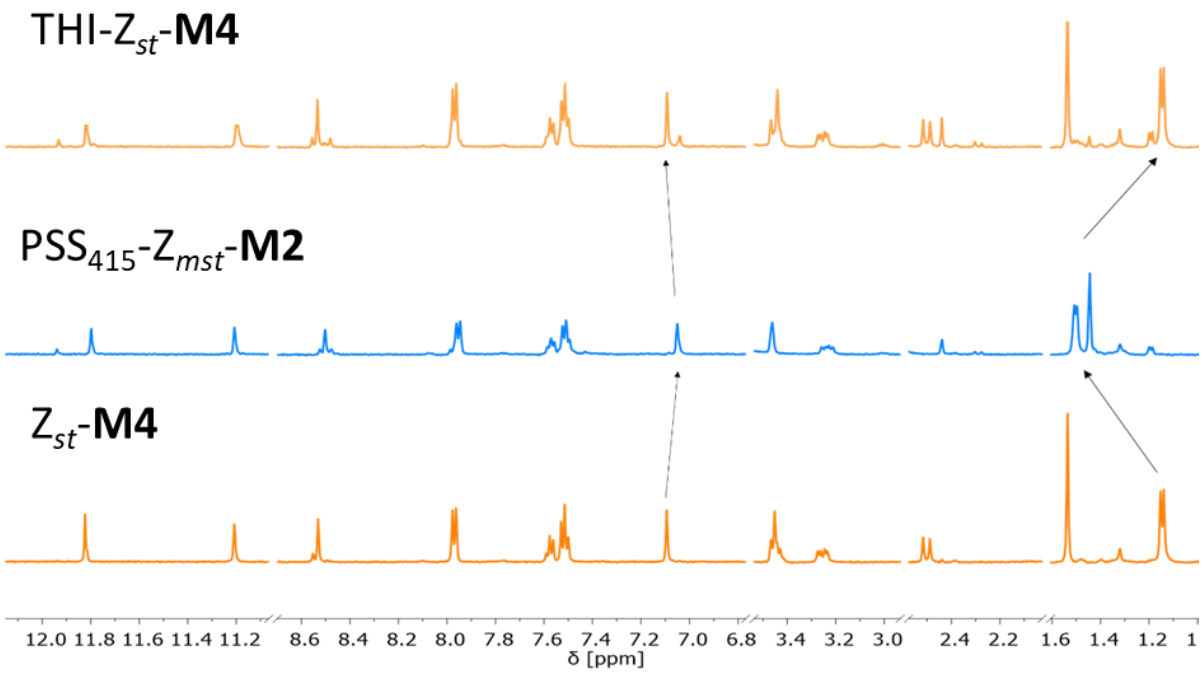

Supplementary Figure 20. **Rotary behaviour of M4 investigated by ^{1}H NMR** (Tetrahydrofuran-D8, 293 K): before irradiation at 293 K (orange, bottom), at PSS (blue, middle) and after THI (orange, top).

8. Photoluminescence of motors

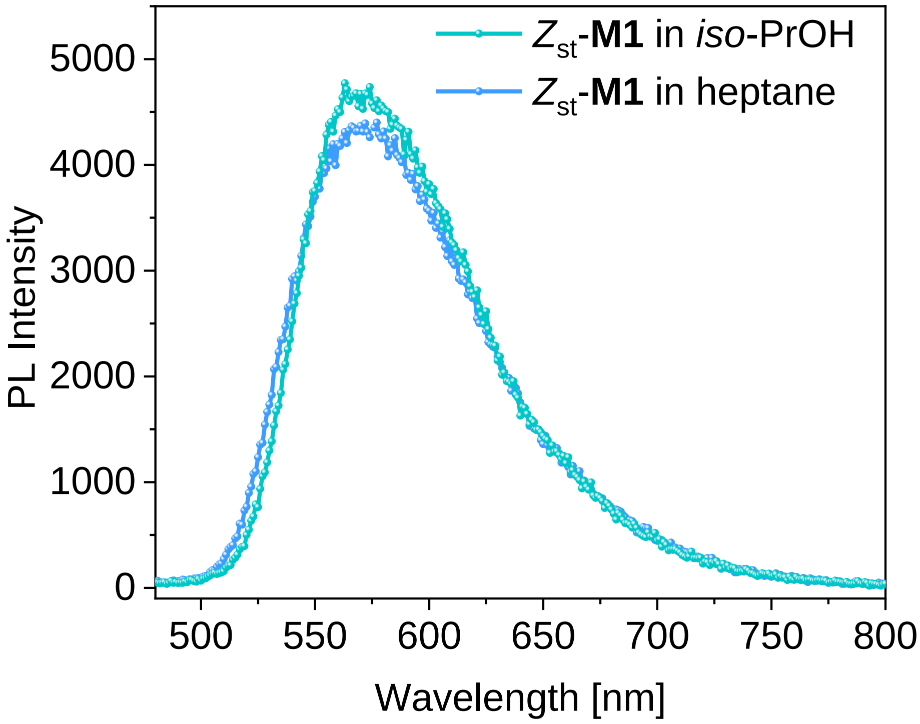

Supplementary Figure 21. **Photoluminesence of Z_{st}-M1 in different solvents** (λ_{ex} = 410 nm), indicating no polarity dependence.

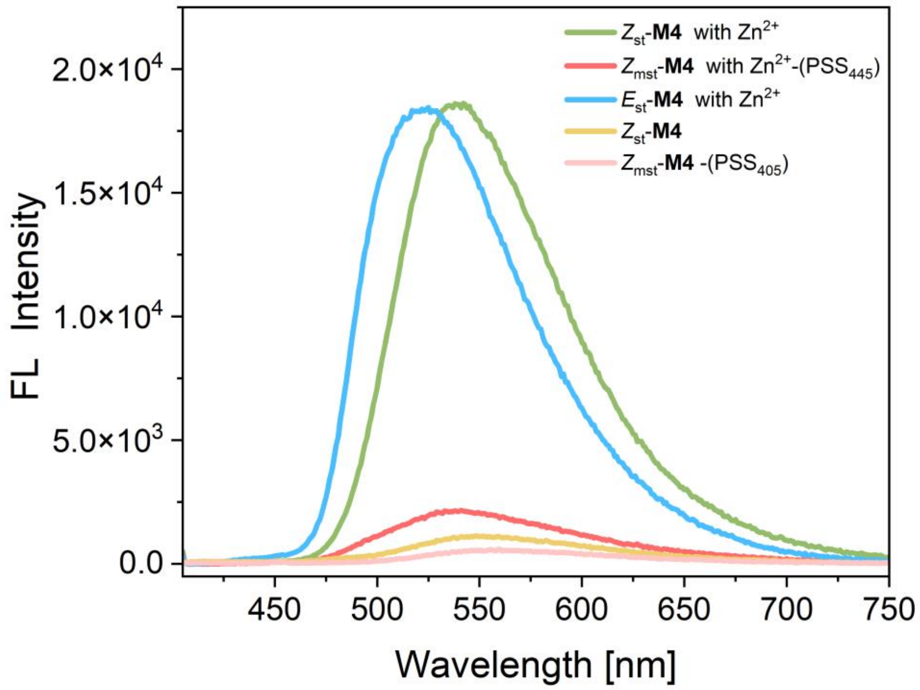

Supplementary Figure 22. **Photoluminescence of M4 with and without Zn^{2+} at different states (λ_{ex} = 400 nm).**

9. Photoisomerization of M4-Zn complex

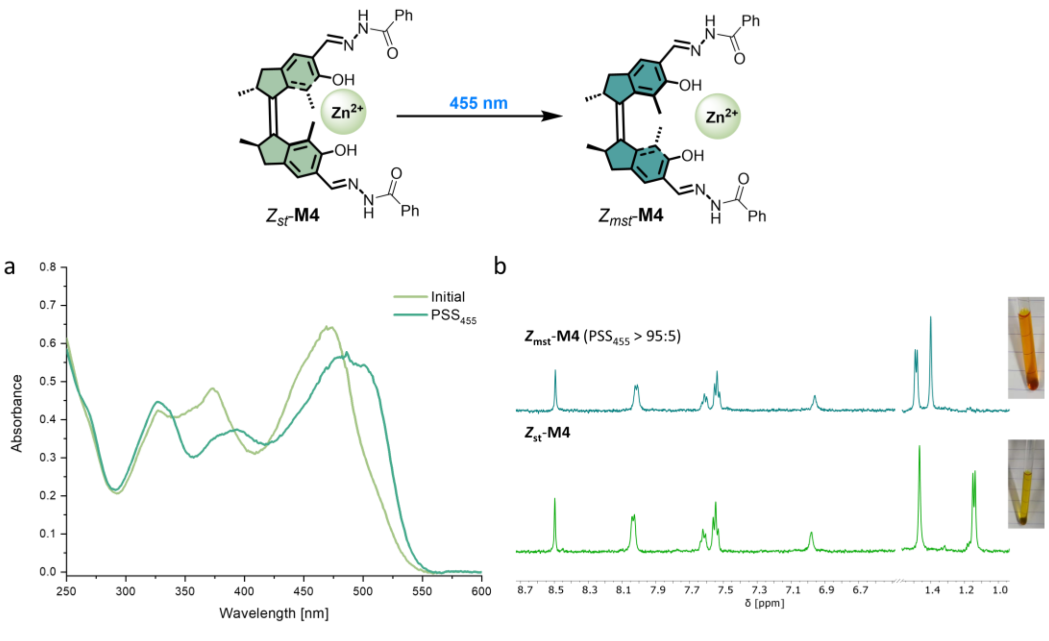

Supplementary Figure 23. **Photoisomerization of M4 with Zn^{2+} monitored by** a. UV-Vis and b. ^{1}H NMR spectroscopy.

10. Titration experiments of M4 with Zn^{2+}

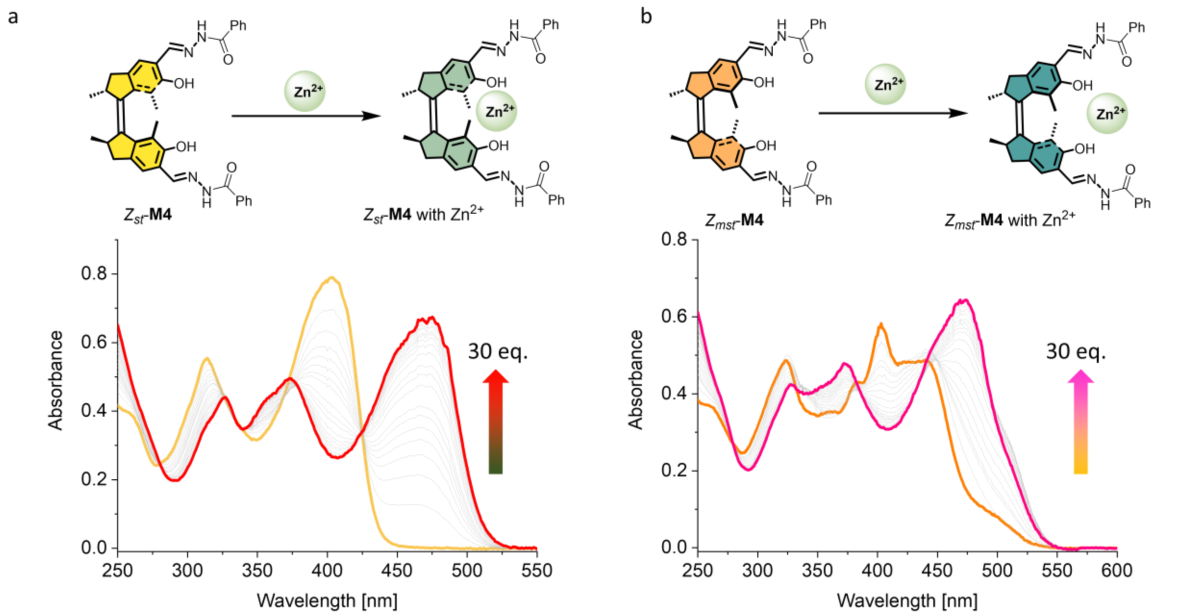

Supplementary Figure 24. **Titration monitored by UV-Vis spectroscopy. a** Z_{st}-**M4** with $Zn(NO_3)_2$ in THF. **b** Z_{mst}-**M4** (PSS_{405}) with $Zn(NO_3)_2$ in THF.

11. Fatigue studies

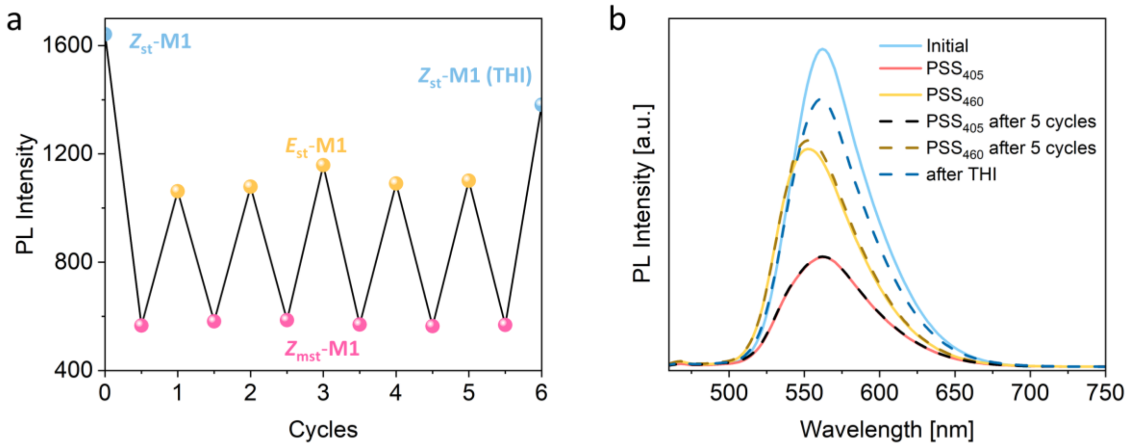

Supplementary Figure 25. **Fatigue studies. a** PL Fatigue study starting from Z_{st}-**M1 to** Z_{mst}-**M1,** then switching between Z_{mst}-**M1** (PSS_{405}) and E_{st}-**M1** (PSS_{460}) for five cycles, and finally heating back to Z_{st}-**M1**. **b** PL spectra of initial Z_{st}-**M1**, Z_{mst}-**M1** (PSS_{405}) and E_{st}-**M1** (PSS_{460}).

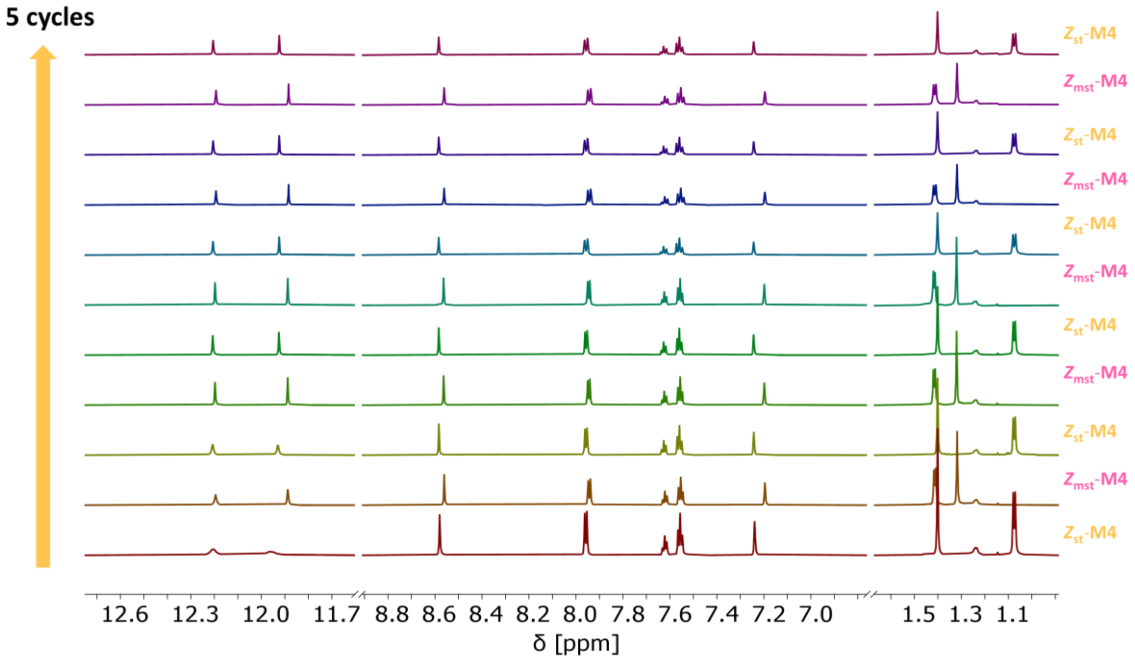

Supplementary Figure 26. **^{1}H NMR Fatigue study starting from Z_{st}-M4 to Z_{mst}-M4 (PSS$_{405}$), and heating back to Z_{st}-M4 for 5 cycles.**

12. Computational Analysis

All structures were pre-screened using the CREST[2] driver (version 2.11) in the xTB 6.3.2 software, using the GFN2-xTB semiempirical level of theory.[3] A selection of conformers was subsequently optimized at the r^2SCAN-3c[4] level of theory, as implemented in the Orca 5.0.4 package,[5] to identify the lowest energy conformation. Final geometry optimizations were done at the r^2SCAN-3c/CPCM(CH_2Cl_2)[6-9] or r^2SCAN-3c/CPCM(DMSO)[6-9] level of theory and the thermochemical data were calculated at the same level of theory. Full TDDFT calculations were performed at the CAM-B3LYP-D3BJ/def2-TZVPP/CPCM(CH_2Cl_2)[6-9] or CAM-B3LYP-D3BJ/def2-TZVPP/CPCM(DMSO) level of theory without the Tamm-Dancoff approximation, considering 30 singlet transitions. The XYZ coordinates of all optimized structures are provided as source data.

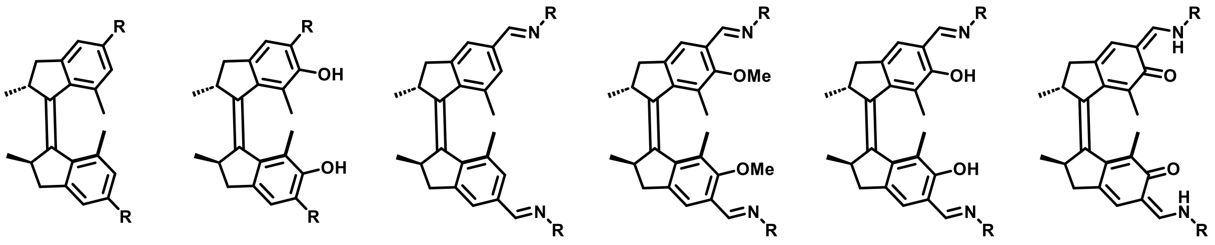

M-oR M-oOH M-H M-OMe M-OH M-NH

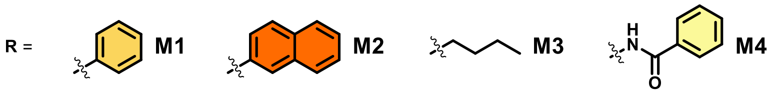

	M-oR	M-oOH	M-H	M-OMe	M-OH	M-NH
**M1**	336	332	379	384	395	441
**M2**	347	342	385	389	407	449
**M3**	308	316	349	353	360	399
**M4**	338	339	379	384	387	-
**M4 (DMSO)**	339	339	381	386	388	-

Supplementary Table 1. Computed absorption wavelengths corresponding to the first transition of the stable *Z* conformations of the salicylidene Schiff-base-derived molecular motors and their related structures. These transitions were also calculated to have the highest oscillator strengths. Calculations were performed at the CAM-B3LYP-D3BJ/def2-TZVPP/CPCM(CH_2Cl_2)//r^2SCAN-3c/CPCM(CH_2Cl_2) or CAM-B3LYP-D3BJ/def2-TZVPP/CPCM(DMSO)//r^2SCAN-3c/CPCM(DMSO) level of theory.

13.NMR and HRMS spectra

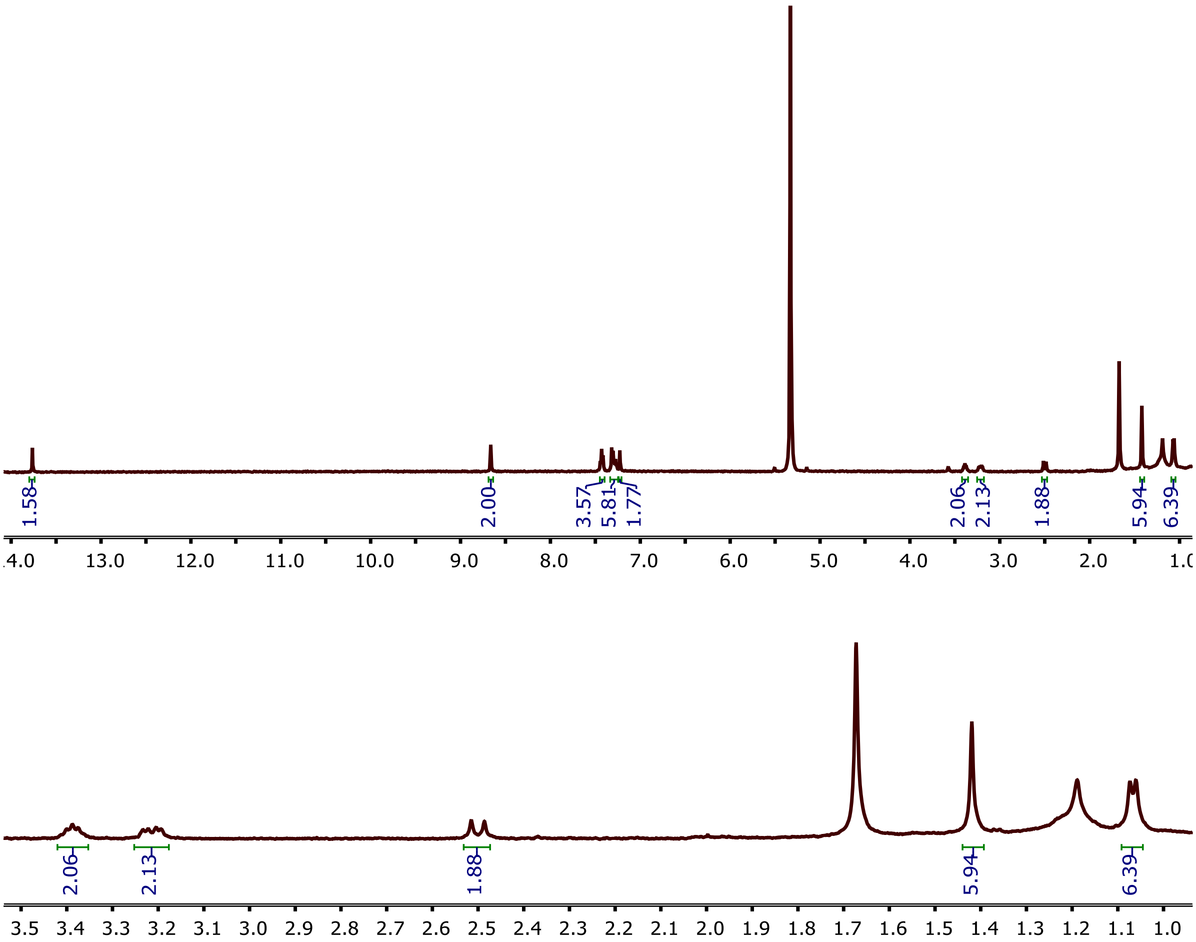

Supplementary Figure 27. ^{1}H NMR (400 MHz) spectrum of ***Z*$_{st}$-M1** measured in CD_2Cl_2 at 25 °C

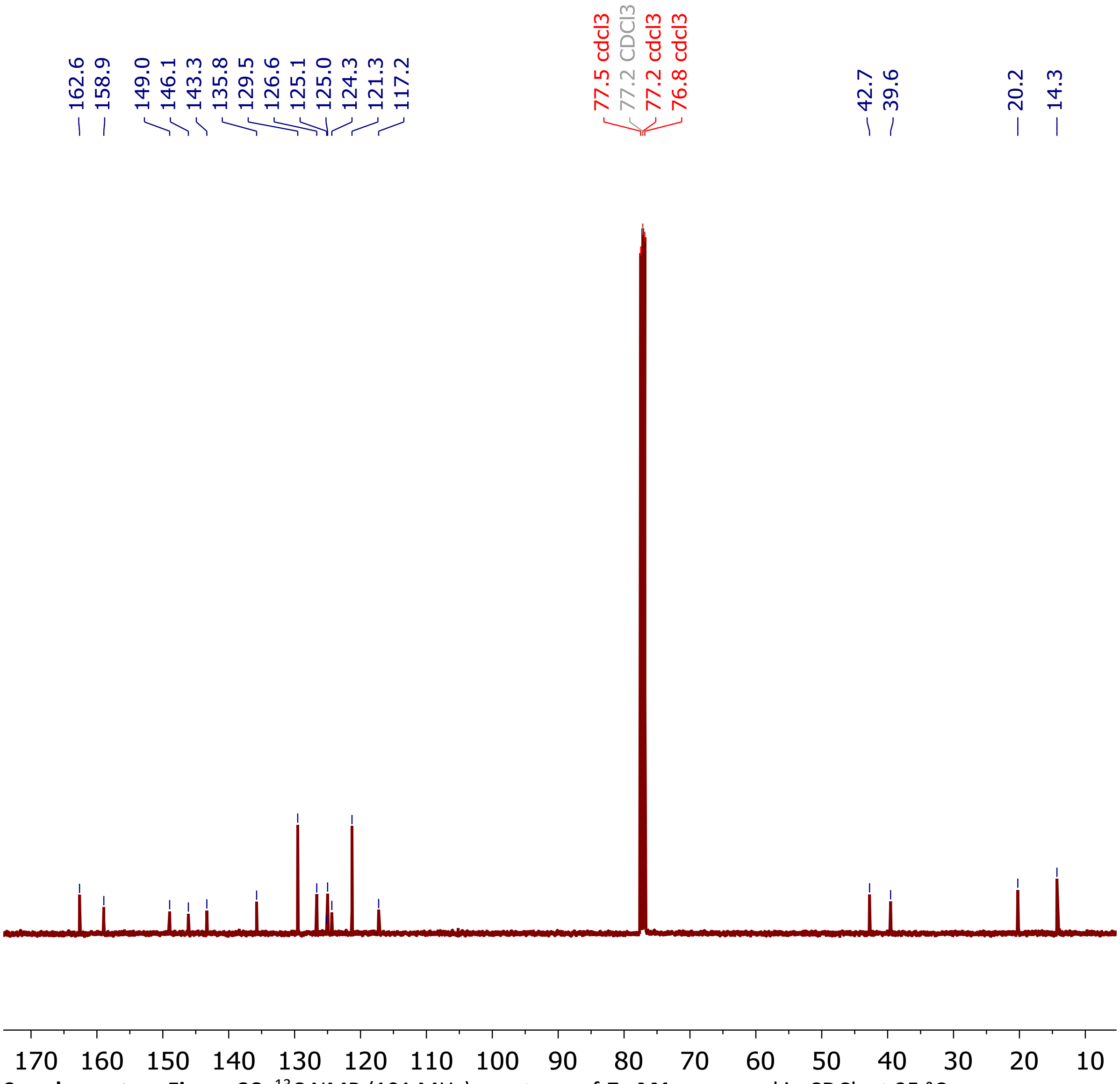

Supplementary Figure 28. ^{13}C NMR (101 MHz) spectrum of ***Z*$_{st}$-M1** measured in $CDCl_3$ at 25 °C

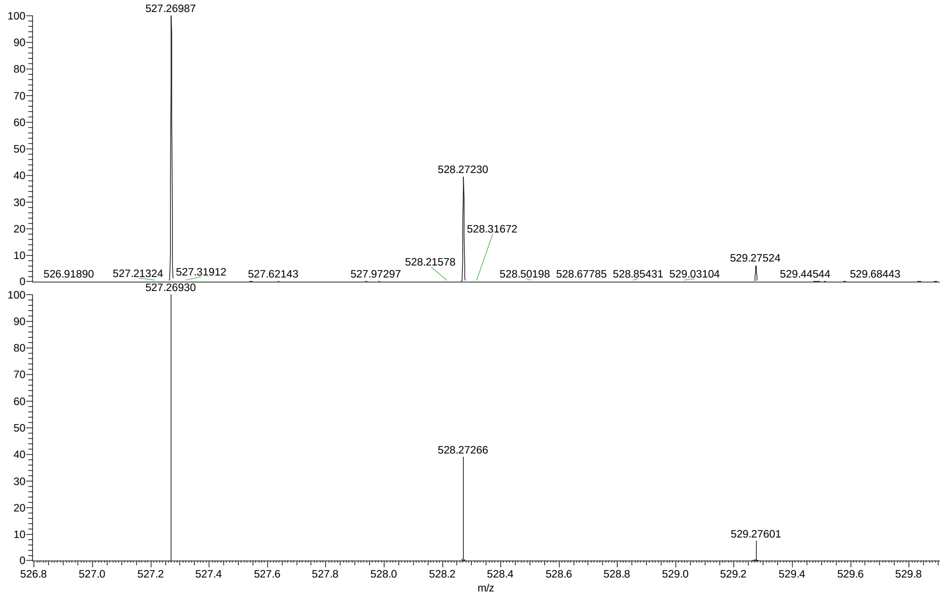

Supplementary Figure 29. HRMS spectrum of ***Z*$_{st}$-M1.**

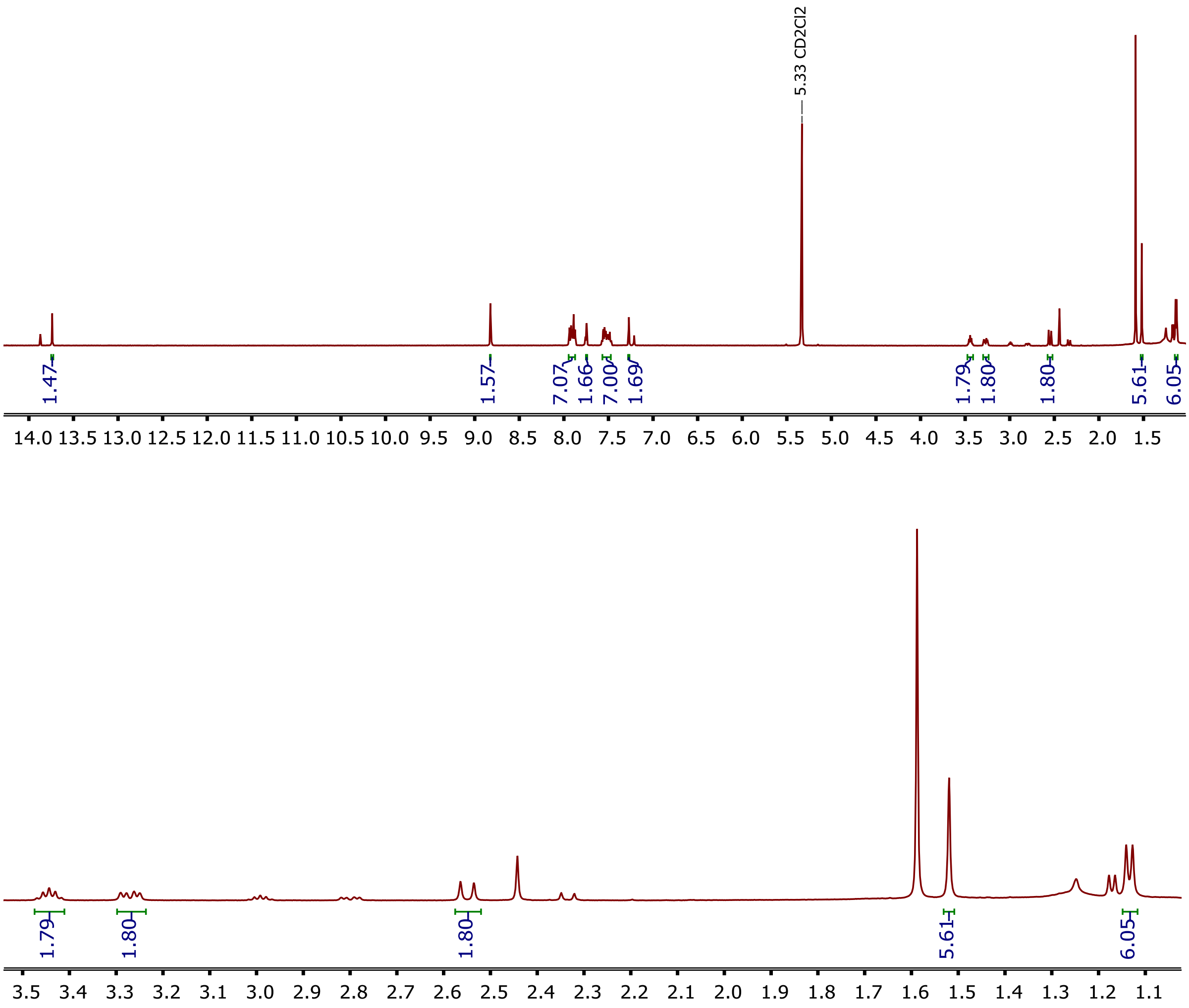

Supplementary Figure 30. ^{1}H NMR (400 MHz) spectrum of ***Z*$_{st}$-M2** measured in CD_2Cl_2 at 25 °C

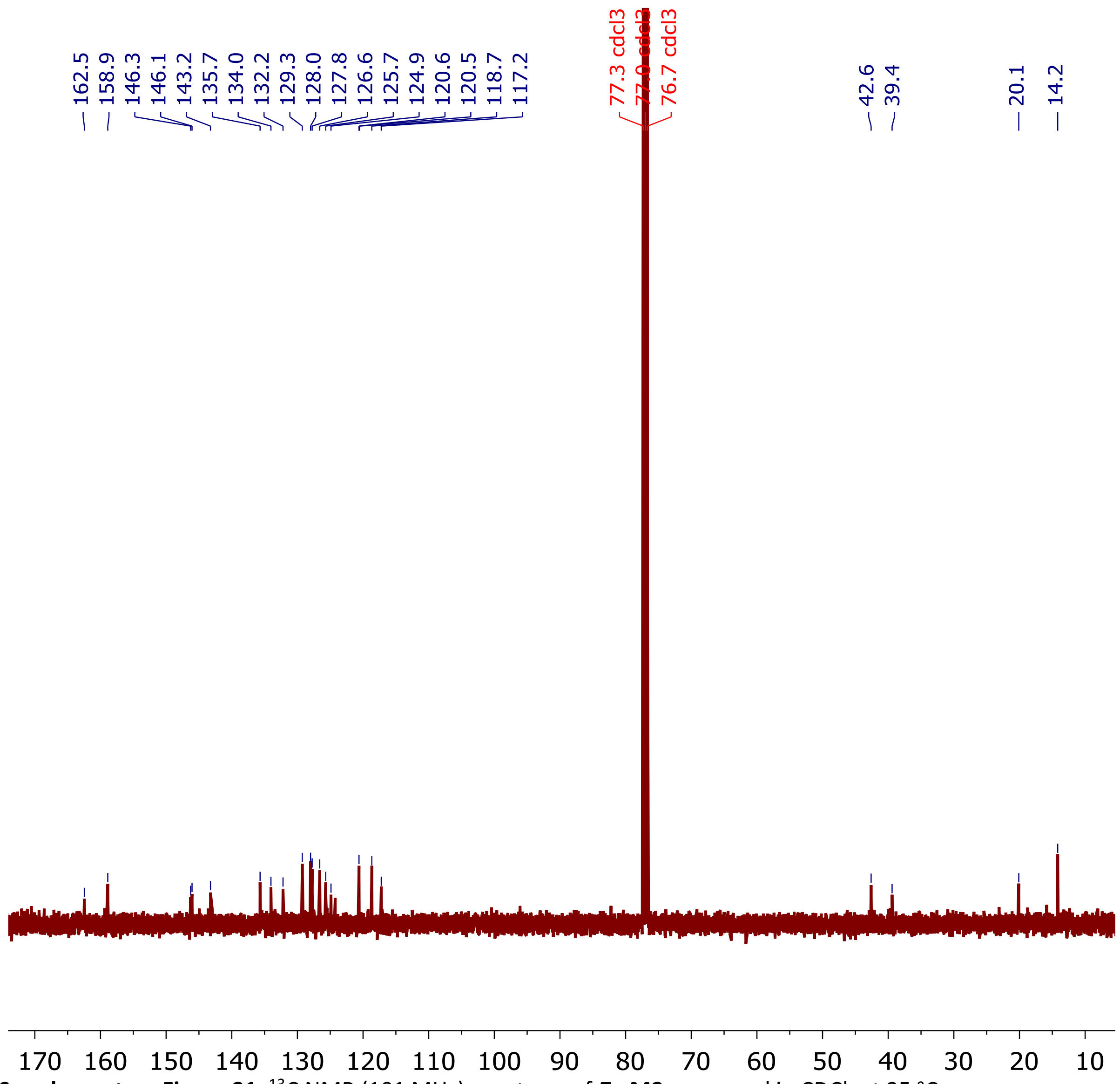

Supplementary Figure 31. ^{13}C NMR (101 MHz) spectrum of ***Z*$_{st}$-M2** measured in $CDCl_3$ at 25 °C

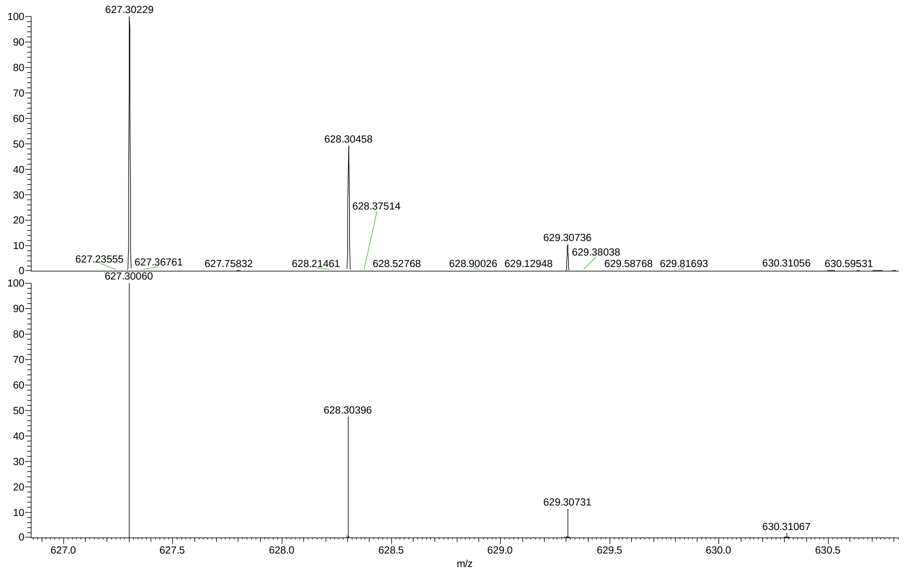

Supplementary Figure 32. HRMS spectrum of ***Z*$_{st}$-M2.**

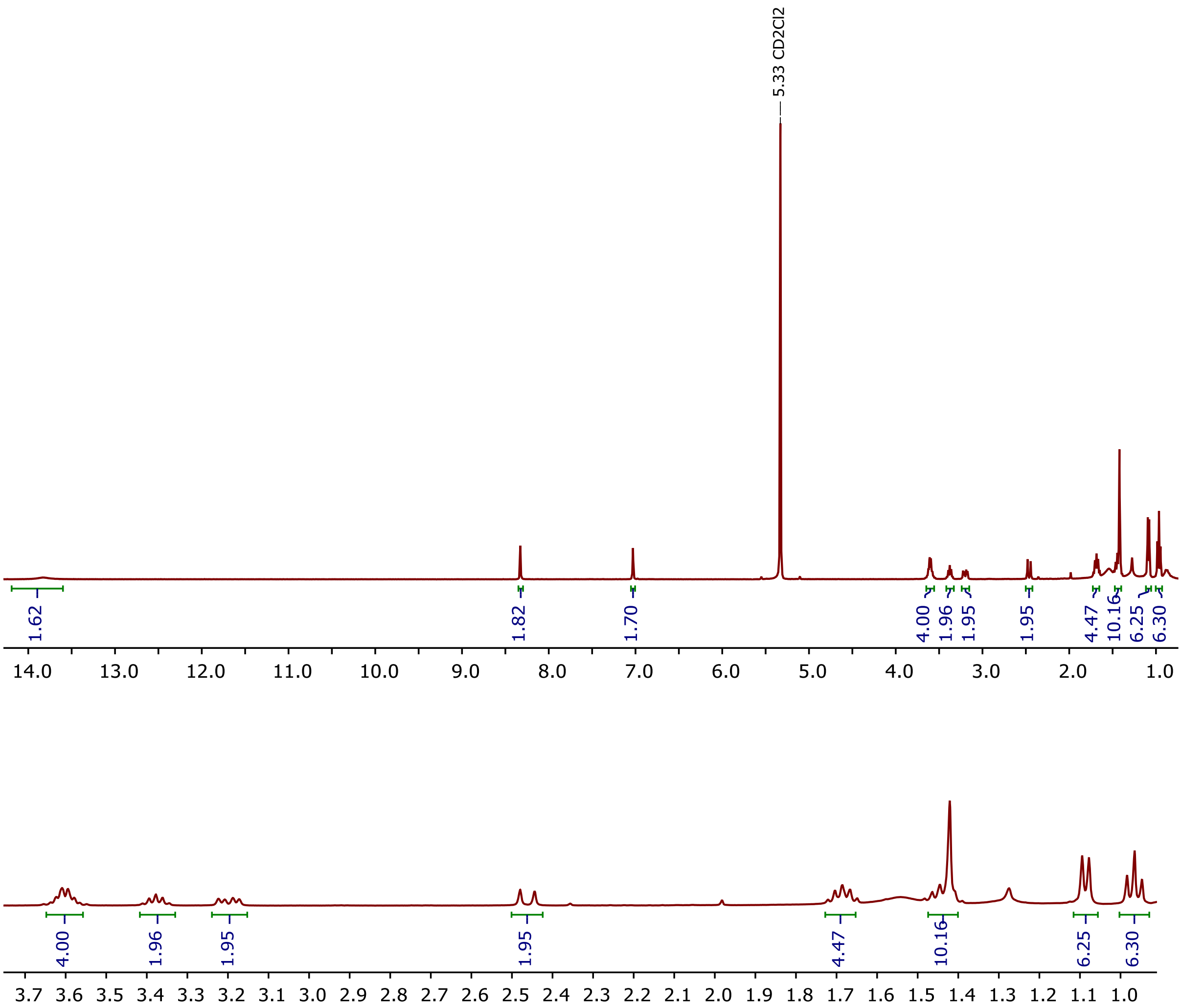

Supplementary Figure 33. ^{1}H NMR (400 MHz) spectrum of ***Z*$_{st}$-M3** measured in CD_2Cl_2 at 25 °C

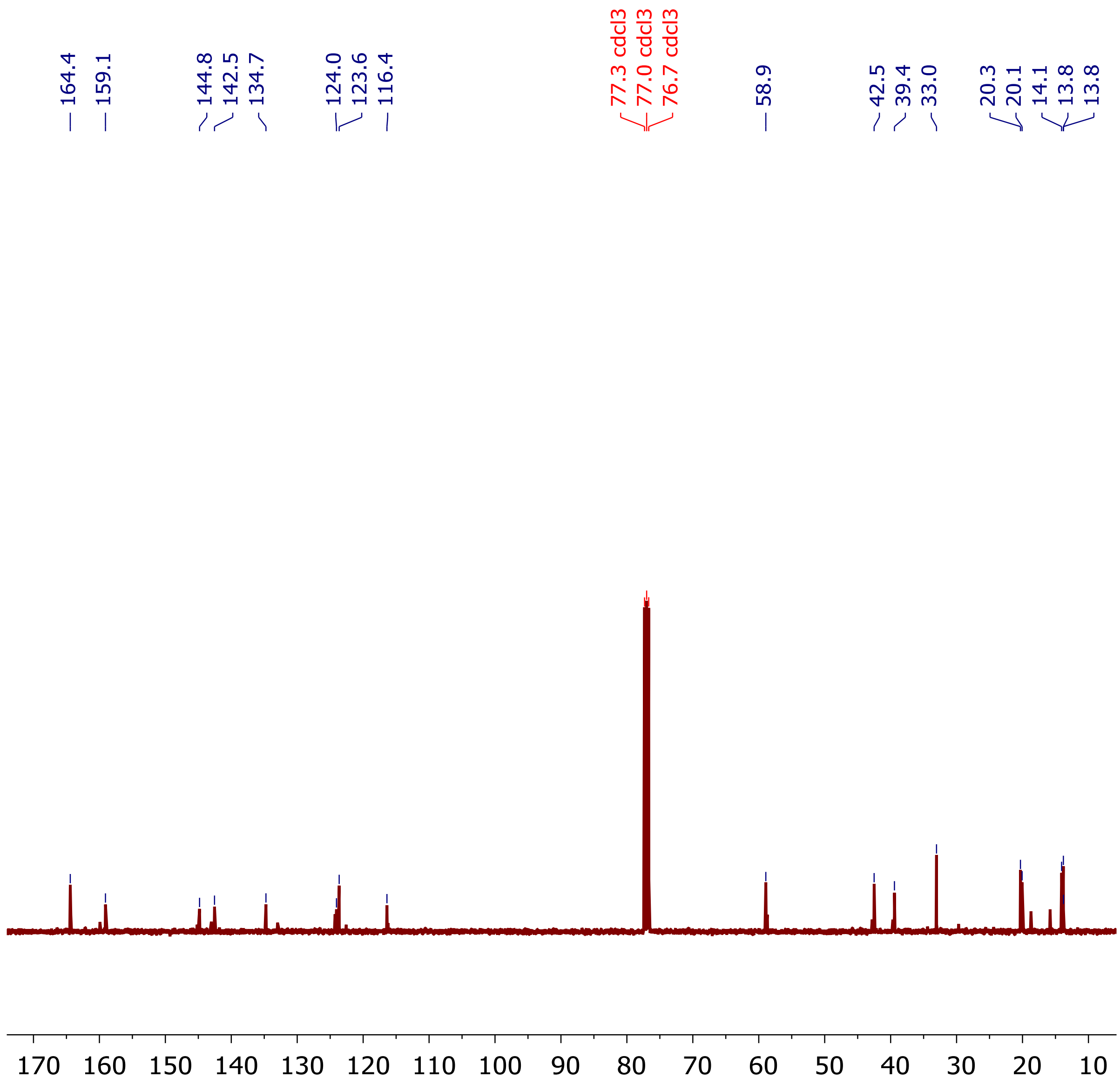

Supplementary Figure 34. ^{13}C NMR (101 MHz) spectrum of ***Z*$_{st}$-M3** measured in $CDCl_3$ at 25 °C

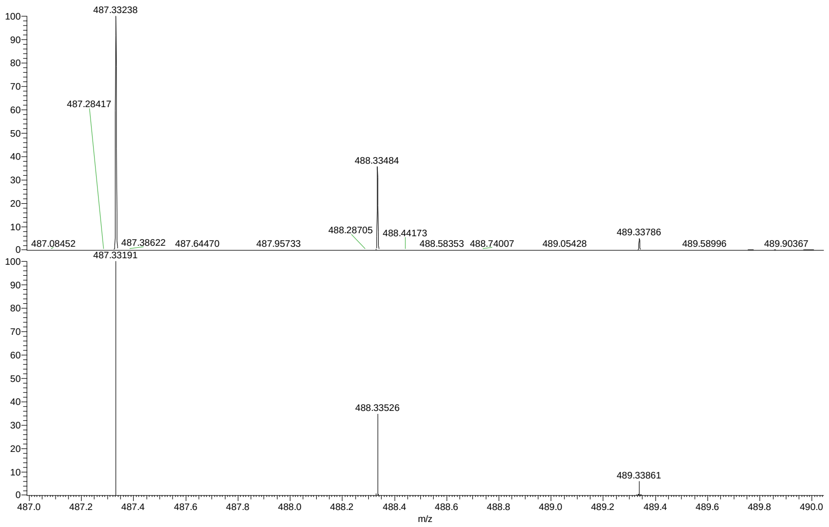

Supplementary Figure 35. HRMS spectrum of ***Z*$_{st}$-M3.**

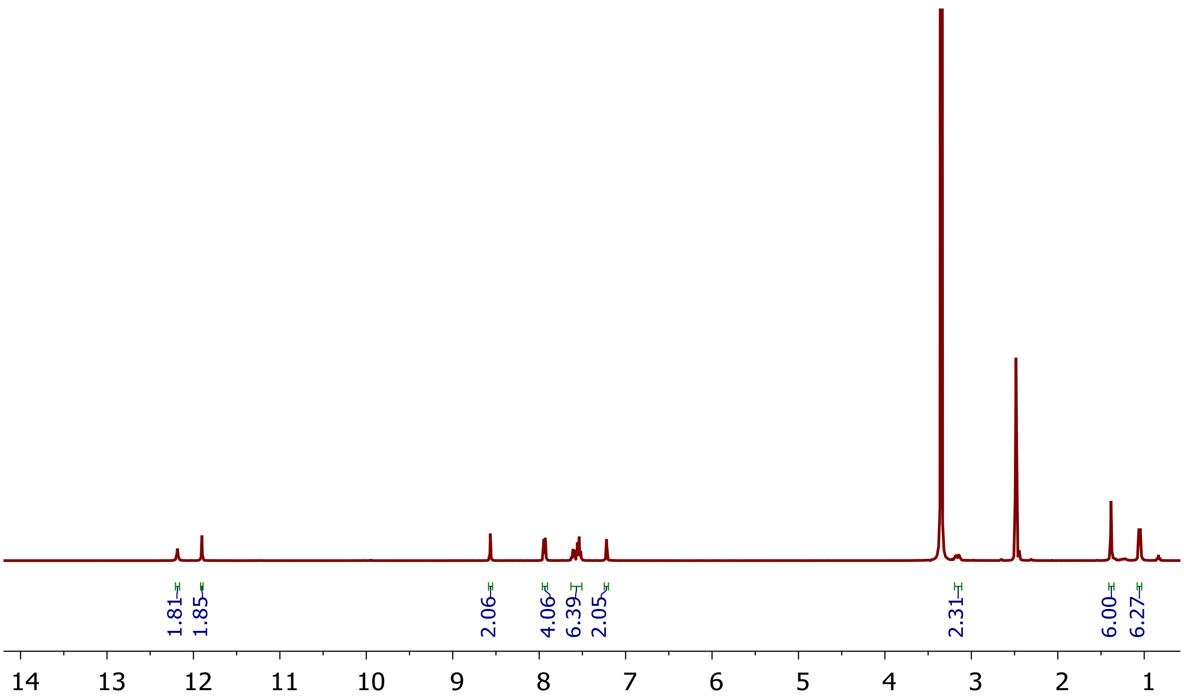

Supplementary Figure 36. ^{1}H NMR (400 MHz) spectrum of ***Z***$_{st}$**-M1** measured in DMSO-d_6 at 25 °C

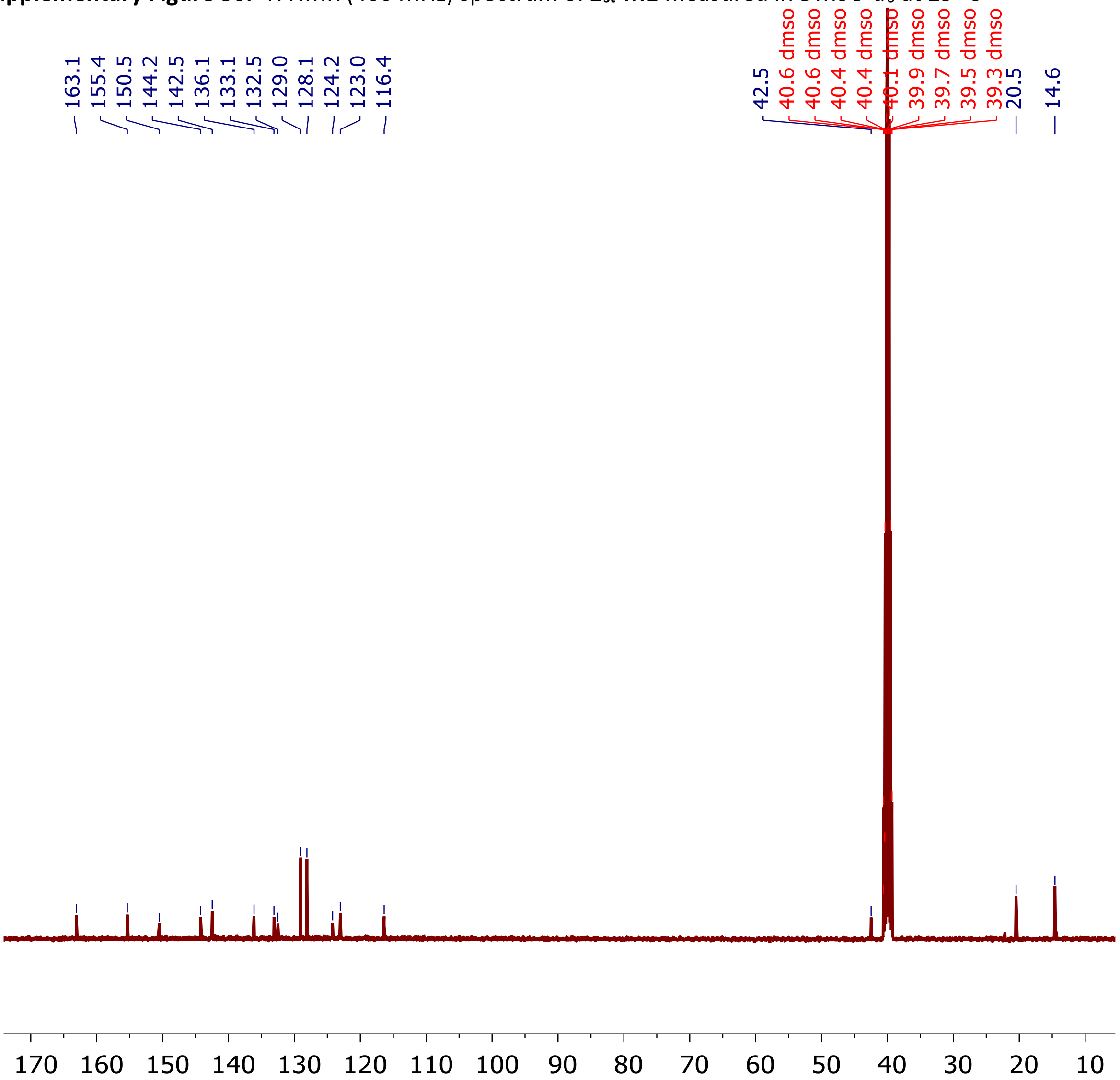

Supplementary Figure 37. ^{13}C NMR (101 MHz) spectrum of ***Z***$_{st}$**-M4** measured in DMSO-$d_6$at 25 °C

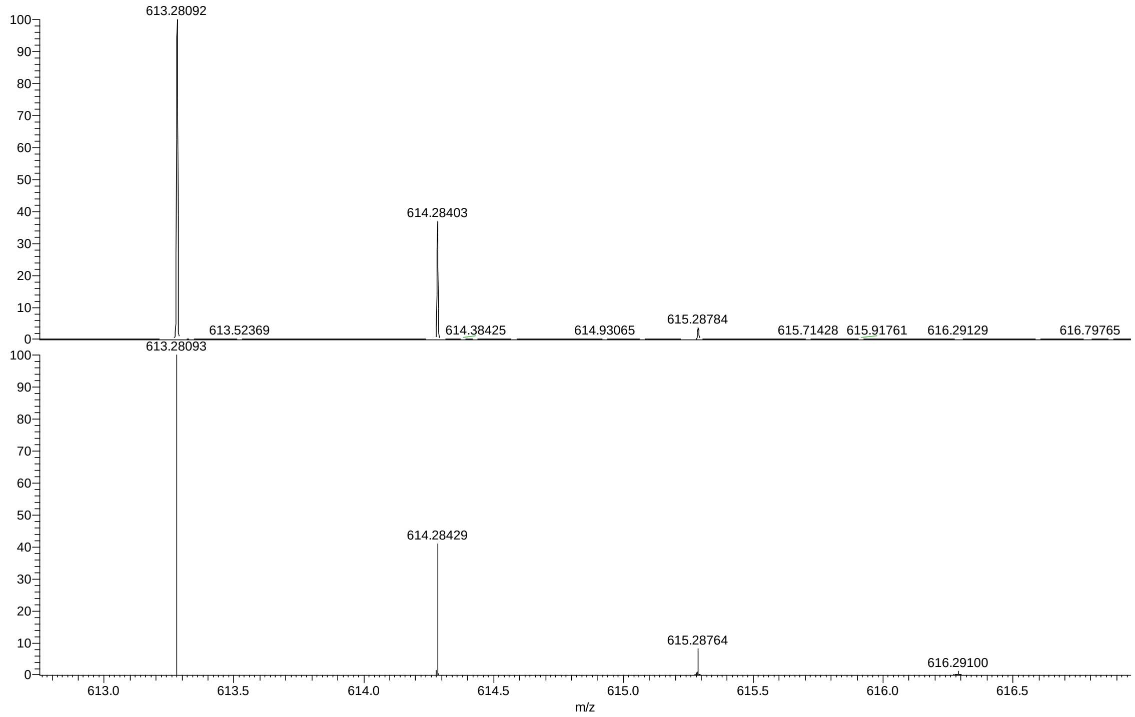

Supplementary Figure 38. HRMS spectrum of ***Z*$_{st}$-M4.**

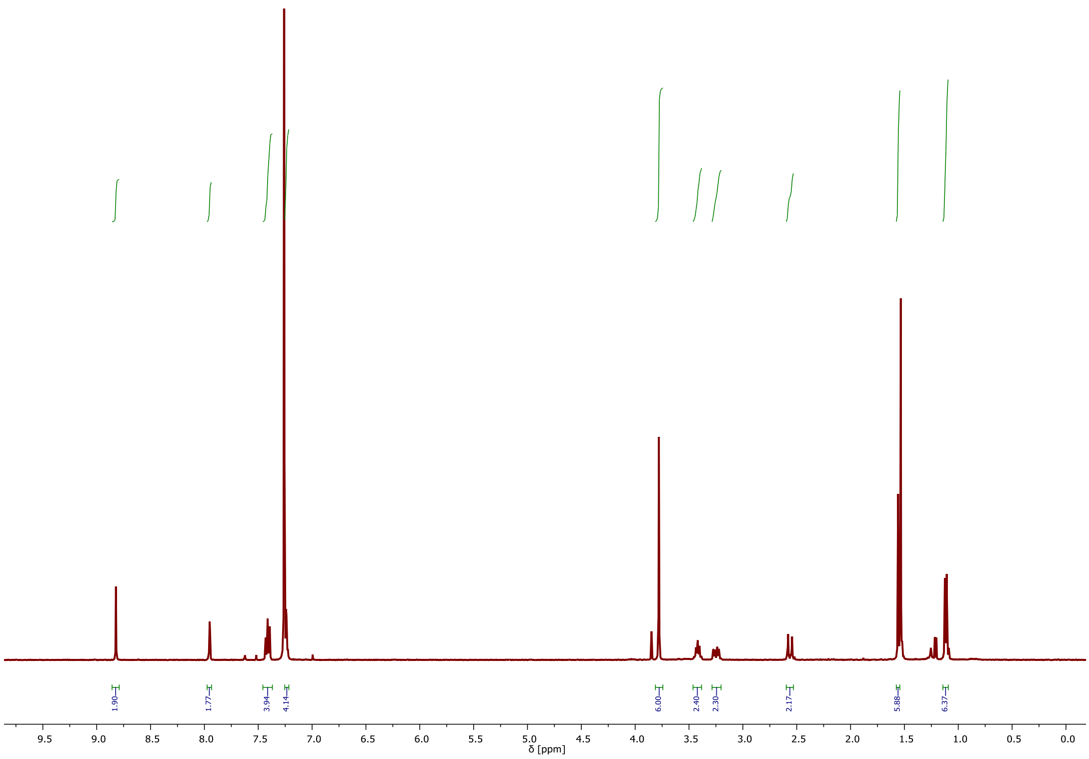

Supplementary Figure 39. ^{1}H NMR (400 MHz) spectrum of ***Z*$_{st}$-M1-OMe** measured in $CDCl_3$ at 25 °C

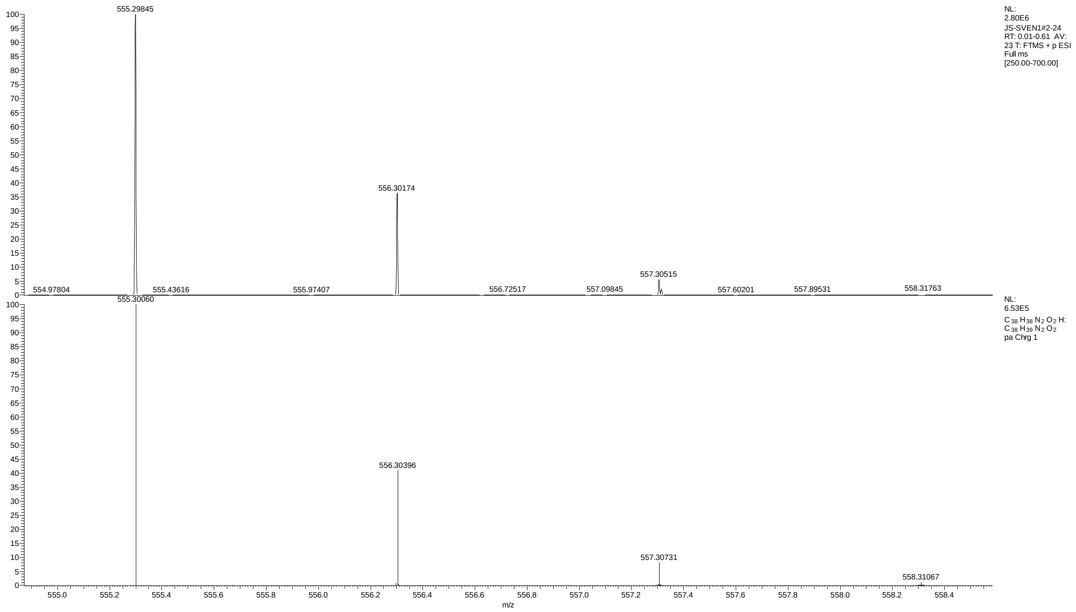

Supplementary Figure 40. HRMS spectrum of ***Z*$_{st}$-M1-OMe.**

Supplementary References

1 Sheng, J. *et al.* Formylation boosts the performance of light-driven overcrowded alkene-derived rotary molecular motors. *Nat. Chem.* (2024). https://doi.org/10.1038/s41557-024-01521-0

2 Pracht, P., Bohle, F. & Grimme, S. Automated exploration of the low-energy chemical space with fast quantum chemical methods. *Phys. Chem. Chem. Phys.* **22**, 7169-7192 (2020). https://doi.org/10.1039/c9cp06869d

3 Bannwarth, C., Ehlert, S. & Grimme, S. GFN2-xTB-An Accurate and Broadly Parametrized Self-Consistent Tight-Binding Quantum Chemical Method with Multipole Electrostatics and Density-Dependent Dispersion Contributions. *J. Chem. Theory Comput.* **15**, 1652-1671 (2019). https://doi.org/10.1021/acs.jctc.8b01176

4 Grimme, S., Hansen, A., Ehlert, S. & Mewes, J. M. r(2)SCAN-3c: A "Swiss army knife" composite electronic-structure method. *J. Chem. Phys.* **154**, 064103 (2021). https://doi.org/10.1063/5.0040021

5 Neese, F., Wennmohs, F., Becker, U. & Riplinger, C. The ORCA quantum chemistry program package. *J. Chem. Phys.* **152**, 224108 (2020). https://doi.org/10.1063/5.0004608

6 Barone, V. & Cossi, M. Quantum Calculation of Molecular Energies and Energy Gradients in Solution by a Conductor Solvent Model. *J. Phys. Chem. A* **102**, 1995-2001 (1998). https://doi.org/10.1021/jp9716997

7 Yanai, T., Tew, D. P. & Handy, N. C. A new hybrid exchange–correlation functional using the Coulomb-attenuating method (CAM-B3LYP). *Chem. Phys. Lett.* **393**, 51-57 (2004). https://doi.org/10.1016/j.cplett.2004.06.011

8 Weigend, F. & Ahlrichs, R. Balanced basis sets of split valence, triple zeta valence and quadruple zeta valence quality for H to Rn: Design and assessment of accuracy. *Phys. Chem. Chem. Phys.* **7**, 3297-3305 (2005). https://doi.org/10.1039/b508541a

9 Grimme, S., Ehrlich, S. & Goerigk, L. Effect of the damping function in dispersion corrected density functional theory. *J. Comput. Chem.* **32**, 1456-1465 (2011). https://doi.org/10.1002/jcc.21759
